# Blade and bladelet production at Hohle Fels Cave, AH IV in the Swabian Jura and its importance for characterizing the technological variability of the Aurignacian in Central Europe

**DOI:** 10.1371/journal.pone.0194097

**Published:** 2018-04-09

**Authors:** Guido Bataille, Nicholas J. Conard

**Affiliations:** Eberhard Karls Universität Tübingen, Institut für Ur- und Frühgeschichte und Archäologie des Mittelalters, Abteilung für Urgeschichte und Quartärökologie, Burgsteige, Baden-Württemberg, Tübingen, Germany; Universita degli Studi di Ferrara, ITALY

## Abstract

Hohle Fels Cave in the Ach Valley of Southwestern Germany exhibits an Aurignacian sequence of 1 m thickness within geological horizons (GH) 6–8. The deposition of the layers took place during mild and cold phases between at least 42 ka (GI 10) and 36 ka calBP (GI 7). We present below a technological study of blade and bladelet production from AH IV (GH 7) at Hohle Fels. Our analyses show that blade manufacture is relatively constant, while bladelet production displays a high degree of variability in order to obtain different blanks. Knappers used a variety of burins as cores to produce fine bladelets. The results reveal a new variant of the Aurignacian in the Swabian Jura primarily characterized by the production of bladelets and microliths from burin-cores. The artefacts from the Swabian Aurignacian are technologically and functionally more diverse than earlier studies of the Geißenklösterle and Vogelherd sequences have suggested. The technological analyses presented here challenge the claim that the typo-chronological system from Southwestern Europe can be applied to the Central European Aurignacian. Instead, we emphasize the impact of technological and functional variables within the Aurignacian of the Swabian Jura.

## 1. Introduction

The Swabian Jura is a region of crucial importance in understanding the origin and development of the Aurignacian. The lowest Aurignacian horizons of the region (e.g., Geißenklösterle, AH III, and Hohle Fels, AH Vb) are among the oldest known assemblages of the technocomplex [1]. These deposits also produced early symbolic artefacts such as organic beads, figurative art objects and bone flutes [2–4]. Hohle Fels Cave in the Ach Valley near Schelklingen exhibits a long Pleistocene stratigraphy with Middle Palaeolithic, Aurignacian, Gravettian and Magdalenian horizons embedded within twelve geological strata [5]. The Aurignacian stratigraphy of 1 m thickness consists of at least seven horizons and sub-horizons (AHs IIIa.1 IIIa, IIIb, IV, Va, Vaa & Vb) within geological horizons GH 6–8. Clusters of ashes, charcoal and artefacts indicate *in situ* zones of human activity [5–9]. The sequence documents an occupational hiatus between the lowermost Aurignacian and the uppermost Middle Palaeolithic horizon (AH VI). Although the Aurignacian deposits at Hohle Fels have slopes as high as 15°, they appear to have experienced little redepostion. In the following, we present results of ongoing technological study of the Aurignacian lithic assemblages from Hohle Fels. Furthermore, after completing excavations of the extensive archaeological horizon AH IV (28 m^2^), which belongs to the upper part of the Aurignacian sequence (GH 7), we can now provide preliminary insights into the lithic assemblage. Detailed technological studies on blade and bladelet cores in connection with a technological attribute analysis of blanks illustrate a previously undescribed operational sequence.

### 1.1 Research focus

The results of our technological analysis of the lithic assemblage AH IV provide new information on the technological variability of the Swabian Aurignacian. Technological investigations of blade and bladelet production demonstrate a specific functional facies within the Swabian Aurignacian (“Hohle Fels facies”). A comparison of this facies with other lithic and organic industries of the region, as well as a review of environmental investigations, will provide a more detailed assessment of the socio-economical variability within the Swabian Aurignacian. It is of special interest to researchers to know whether the Swabian Aurignacian exhibits specific functional and diachronic trends. In this paper, we will investigate this question with the example of the Hohle Fels AH IV lithic assemblage. We also will discuss this assemblage in the context of the Western Central and Western European Aurignacian. In future studies, we hope to investigate the impact of environmental, functional and cultural variations on the Swabian Aurignacian.

## 2. Materials

### 2.1. Geographical situation

**Fig 1:**
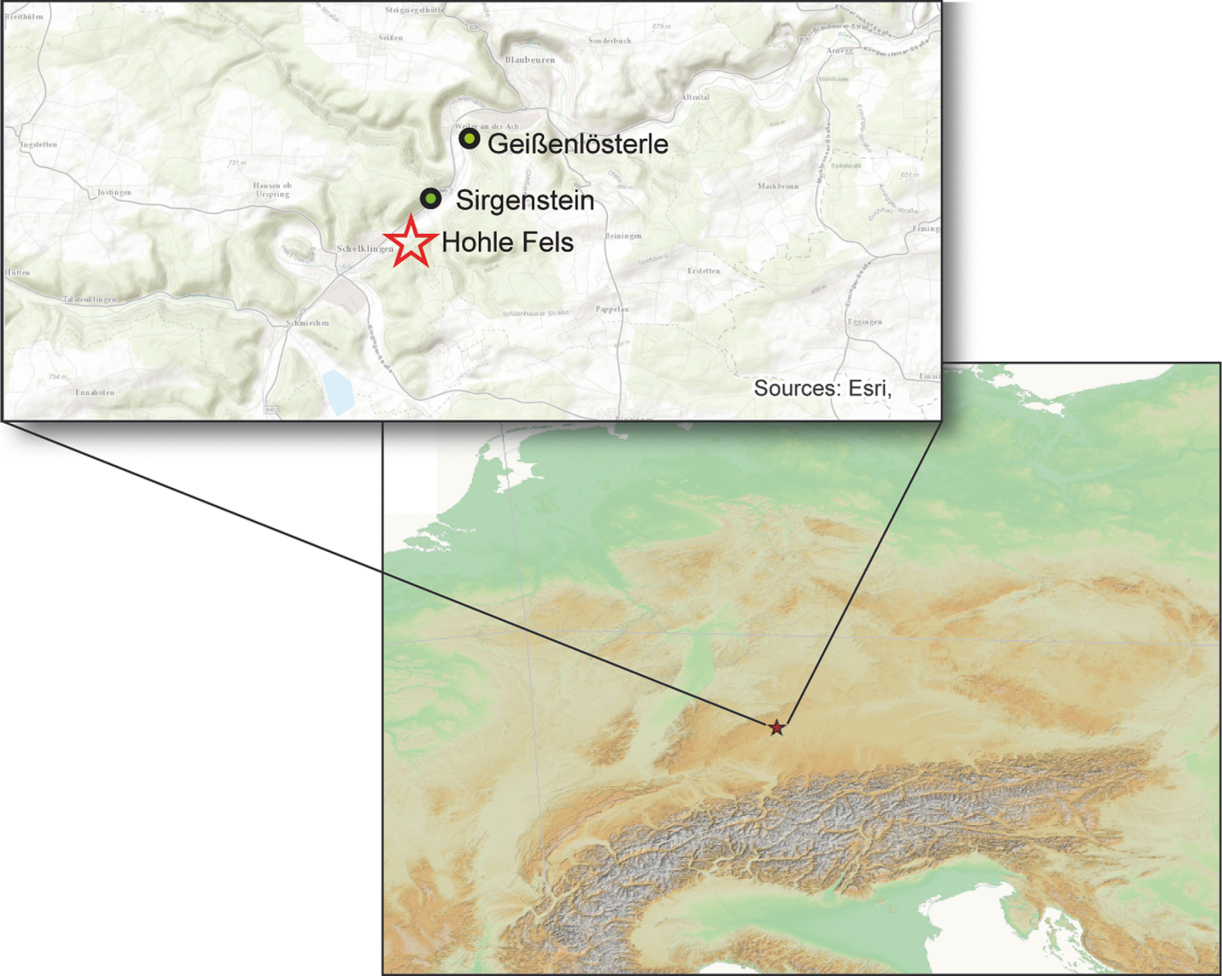
Hohle Fels. Map of the Cave site in the Ach Valley of the Swabian Jura, Southwestern Germany.

**Fig 2:**
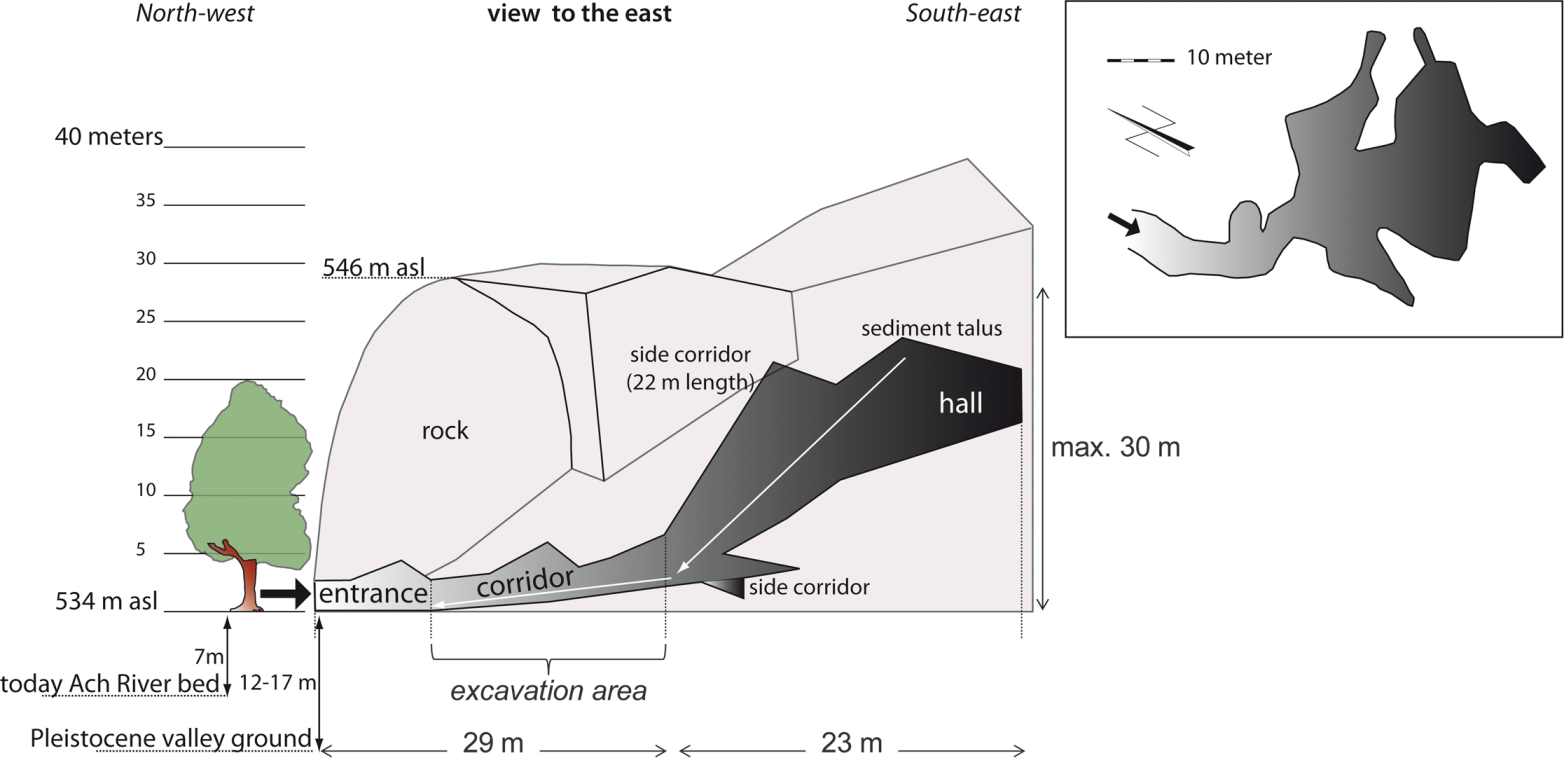
Section through Hohle Fels Cave.

The valleys of the Lone and the Ach, tributaries of the Danube, exhibit important Pleistocene sequences, which yield archaeological horizons of the late Middle and Upper Paleolithic. The numerous Aurignacian horizons in the caves of Geißenklösterle, Hohle Fels and Sirgenstein in the Ach Valley, as well as Vogelherd, Hohlenstein-Stadel and Bockstein in the Lone Valley, have been a focus of scientific interest for decades (Fig 1). Hohle Fels Cave is situated in the Ach Valley, about 1.5 km east of the town of Schelklingen and approximately 20 km west of the city of Ulm. The cave entrance opens towards the northwest, about 7 m above the bank of the present river on the southern face of a Karstic massive, at a height of 534 m a.s.l. [5] (Fig 2). The Ach Valley is presently filled with fluvial sediments, while during the Late Pleistocene the river valley was about 5–10 m lower than it is today.

The main hall of the cave is about 23 m long with a maximum height of 30 m. The main excavation area is situated in a corridor with a length of 29 m that connects the entrance with the main hall.

### 2.2. Research history of the Hohle Fels excavations

Between 1870 and 1871 Oscar Fraas and Theodor J. Hartmann conducted the first scientific excavations in the cave, documenting rich paleontological material as well as cultural material from the Upper Paleolithic [10] (Fig 2). R. R. Schmidt studied the archaeological finds and published them in his seminal monograph from 1912 [11]. After a long break, the Tübingen prehistorian Gustav Riek conducted further excavations from 1958 to 1960 but never published his results [12].

More comprehensive excavations were conducted over multiple seasons between 1977 and 1996 in the entrance corridor and in the main hall under the direction of Joachim Hahn, who planned to use the stratigraphic sequence from Hohle Fels as a point of reference for his excavation at the neighboring site of Geißenklösterle. During these field seasons his team began excavating the site's Magdalenian and Gravettian horizons. Since Hahn's death in 1997, N. J. Conard has directed 21 seasons of excavation at Hohle Fels. This excavation has documented a ca. 5-meter thick stratigraphic sequence reaching bedrock.

### 2.3. Stratigraphy, chronological and environmental context of the Aurignacian deposits (GHs 6, 7 & 8)

Continuous sedimentation took place during the deposition of the late Middle Palaeolithic (GH 9) and early Aurignacian horizons (GH 8) [5]. In contrast to Geißenklösterle, the sequence shows no sign of erosional events between the Middle and the Upper Palaeolithic horizons. The rear of the cave contains a large cone of sediments accumulated via a chimney and cracks in the roof. Downslope movement of these calcareous and phosphatic clay-rich deposits in the form of soil aggregates combined with loess and limestone rubble from the roof and walls of the cave led to the formation of the archaeological strata (Fig 2) [5]. Frost-related features in GH 7 indicate a marked cold-phase during the deposition of that geological horizon. Anthropogenic processes are indicated by lithic and organic artefacts, dumps, and combustion features.

**Fig 3:**
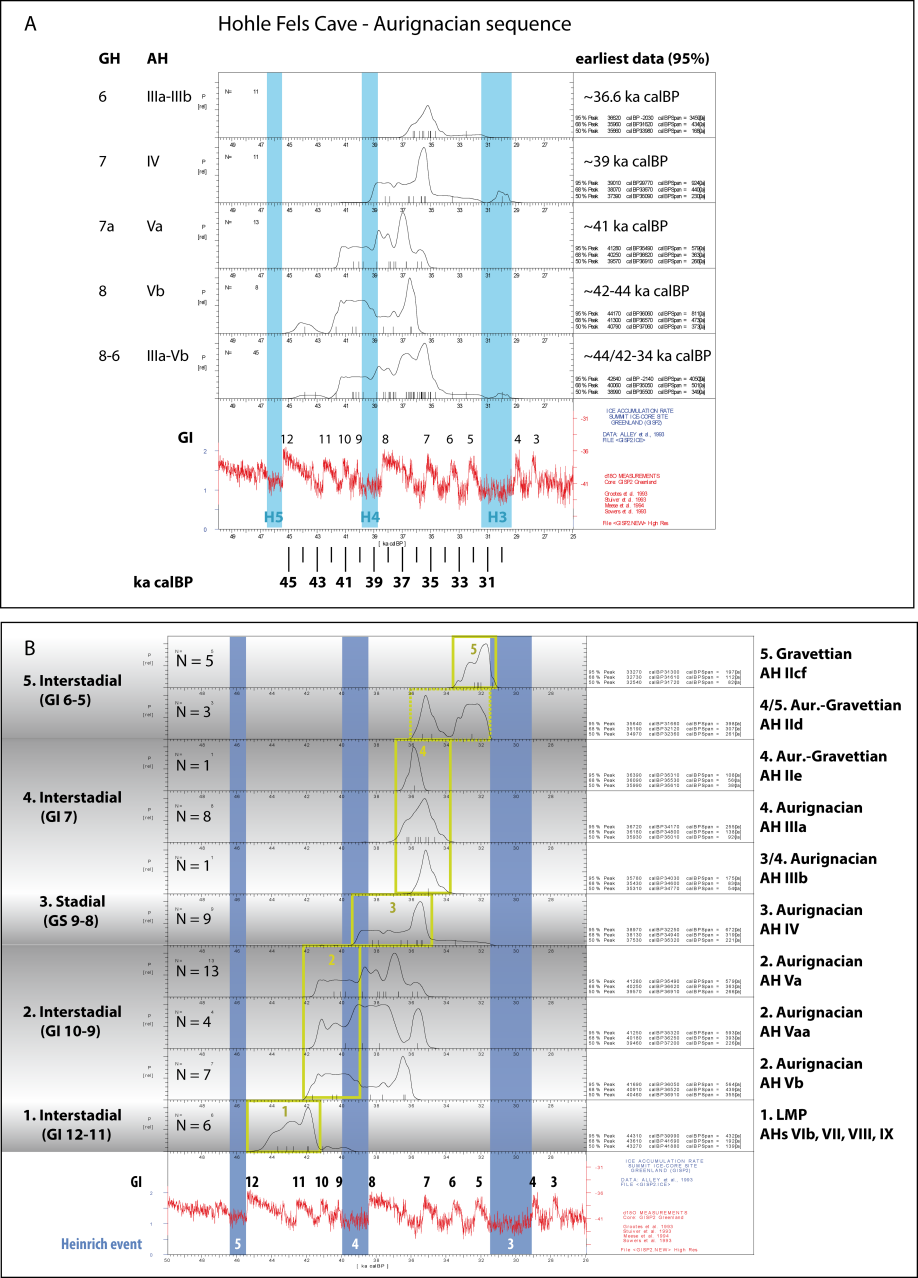
(A) Calibrated radiocarbon ages of the Aurignacian stratigraphy of Hohle Fels Cave. Calibrated with Calpal-Hulu [13]. (B) Radiocarbon data of the Aurignacian stratigraphy of Hohle Fels Cave including its upper and lower boundaries. Calibrated with Calpal-Hulu [13]. The yellow borders indicated the most appropriate time-span of the respective archaeological horizons in relation to environmental information and absolute calibrated data of neighbouring horizons.

Together with the Aurignacian horizon AH III of Geißenklösterle, the lower Hohle Fels Aurignacian assemblages are among the oldest examples of this technocomplex [1]. The lowermost inventories AH Va and Vb, which are embedded within the upper portion of GH 8, yielded the oldest calibrated ages between 44 ka (KIA 16034) and 42 ka calBP (KIA 18880 & OxA-19779) (Fig 3A). Disregarding the oldest date of 44 ka calBP, the radiocarbon dates from AH Vb indicate calibrated ages starting around 41.7 ka calBP (95% peak). This age is in agreement with the upper chronological boundary of the Middle Palaeolithic (GH 7, AH VI). According to radiometric data, the end of the Middle Palaeolithic of Hohle Fels lies between 39.9 and 44.2 ka calBP (95% peak) with the highest probability between ca. 44 and 42 ka calBP (Fig 3B). Lower Aurignacian horizons Va and Vb (GH 8) are correlated with a warm phase, which was less developed than in the uppermost Middle Palaeolithic horizon of AH VI (GH 9). Based on micromorphological studies, C. E. Miller concluded that bearers of the Aurignacian “*arrived in the Ach Valley during a warm period about 40 kyr BP and that this warm period was subsequently followed by a cold period*” [5]. Palaeobotanical data of Hohle Fels suggest a tundra dominated by pine with boreal elements during the lower Aurignacian and an increase of willow indicating a shrub tundra during the upper Aurignacian (GH 6) [12]. This view is supported by micromorphological studies indicating “*increasingly colder conditions in the form of frost-related features and decreasing degree of phosphatization*” [12].

Micro-morphological studies indicate that the last Middle Palaeolithic occupation in the Ach Valley took place under relatively warm and wet conditions [5]. The Aurignacian settlement of Hohle Fels began during the formation of the upper portion of GH 8 in a relatively warm climatic phase that was nonetheless cooler than the final phase of the Middle Palaeolithic [5]. Conditions became increasingly colder during the continuous deposition of GH 8 and GH 7. Aurignacian horizons Va, Vaa and Vb are embedded within a soil (*Böckingerboden*), which might also be represented by GH 17 of Geißenklösterle [5]. In Geißenklösterle Cave the earliest Aurignacian starts during a cold period in GH 15, around 42.5 ka calBP [5]. In the event that the Böckingerboden is present below the Aurignacian horizons from Geißenklösterle Cave, the lower Aurignacian assemblages from Hohle Fels should precede the onset of the Aurignacian at Geißenkösterle. As at Geißenklösterle, largely sterile sediments separate the latest Middle Palaeolithic (GH 9) and earliest Aurignacian (GH 8, upper section) occupations of Hohle Fels (GH 8, lower section). Accordingly, these largely sterile deposits of both stratigraphies might belong to the same chronological period [5]. The Aurignacian of Hohle Fels ends within a marked warm-phase in GH 6a-GH 3db, which corresponds to another, but less developed soil formation (Lohnerboden) and might correlate with GH 10 at Geißenklösterle.

Aurignacian horizon IV, which is the focus of this paper, accumulated during a cold phase [5, 12]. The maximum mean age of about 39 ka calBP (95% peak) might suggest a correlation with the end of the marked cold period of the Heinrich-4-event; Riehl et al. [12] correlate it with the cold phase subsequent to GI 8, which is in agreement with the minimum mean age of the horizon (Fig 3A). Overlying archaeological horizon AH IIIb seems to belong to the end of the same cold period, while the uppermost Aurignacian horizon AH IIIa was accumulated under warm and moist conditions with a chronological peak around 36.7–34.2 ka calBP (95% peak). The two "transitional" horizons IId-e, which exhibit a mixture of Aurignacian and Gravettian technological features, seem to fall into the same climatic period and range between 35.6 and 31.3 ka calBP (95% peak).

Regarding the chronological boundaries between the horizons, the succession from the late Middle Palaeolithic (AH VI-XI) to the Aurignacian-Gravettian "transitional" period (AH IId-e) can be divided into four chronological groups (95% peak) (Fig 3B). The late Middle Palaeolithic horizons range between 44.3 and 40 ka calBP, the lower Aurignacian horizons AH Va-Vb range between 41.7 and 39 ka calBP, the upper Aurignacian horizons of AH IIIa-IV range between 39 and 36 ka calBP. The lower “transitional” Aurignacian-Gravettian assemblage AH IIe falls into the same range (36 ka calBP), while chronologically “transitional” horizon IId corresponds to the lower Gravettian range, which starts around 34–32 ka calBP (AH IIcf).

To conclude, calibrated ages of 57 radiocarbon dates from Hohle Fels indicate a minimum age of the Aurignacian between 42 to 36 ka calBP. Considering the maximum age of AH Vb of 44 ka cal BP (95% peak), the beginning of the Aurignacian occupation might have been 2000 calendar years earlier. This observation is in agreement with more recent dating projects on early Aurignacian assemblages of Central Europe [1, 14, 15]. The Aurignacian of Hohle Fels occurs during a warm phase (GI 10) prior to Heinrich event 4 (GH 8-upper part: AHs Vb and Va), is preceded by a marked cold phase (GHs 7/6-lower: AHs IV and IIIb) and ends during a warm phase (GI 7) prior to Heinrich event 3. Further investigations will confirm or refute the validity of this observation for the Aurignacian in southern Central Europe.

## 3. Methods

**Fig 4:**
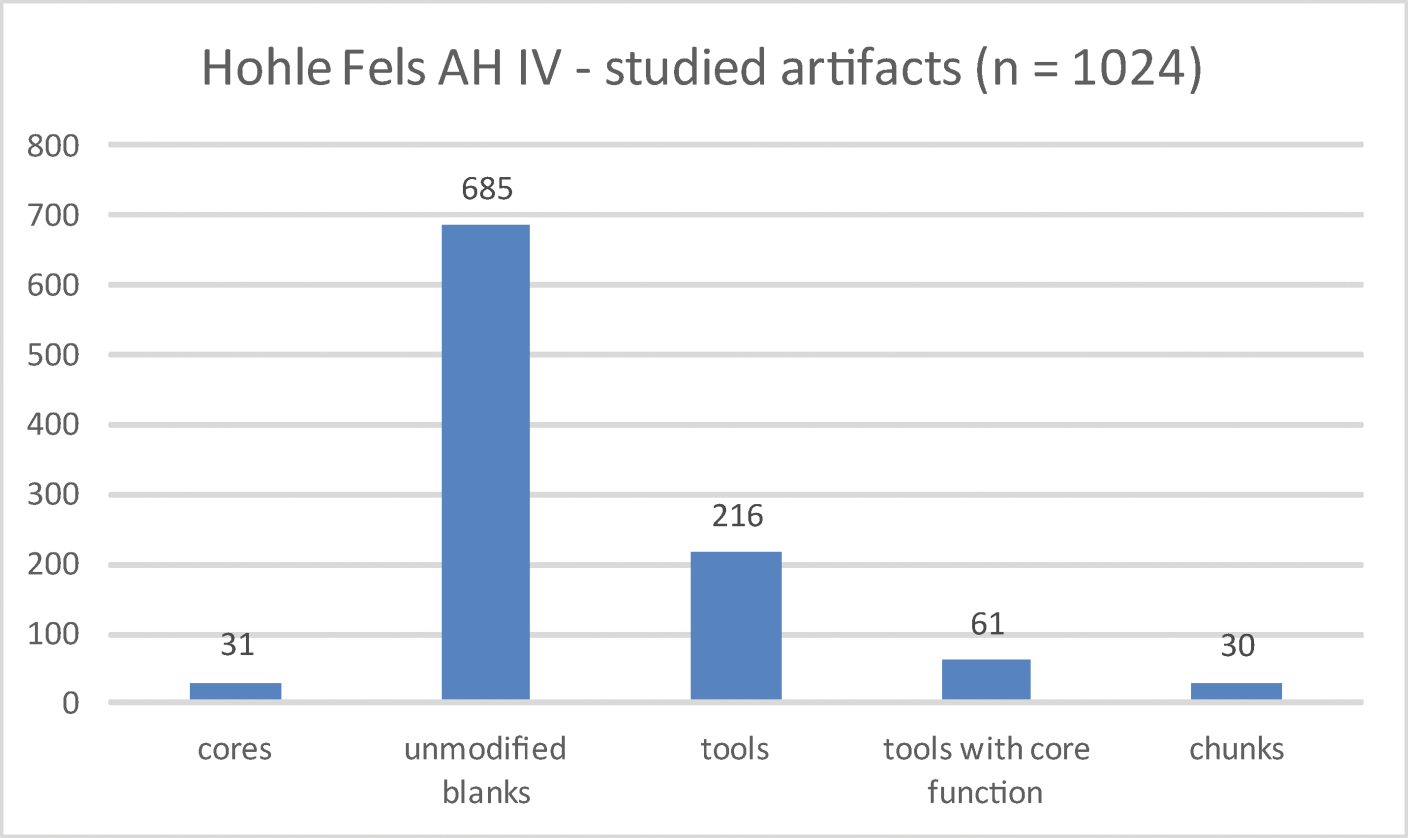
Hohle Fels, AH IV. Analyzed artefacts.

Technological investigations of blade and bladelet cores are the focus of this study. In order to characterize these reduction concepts, we analyzed the operational sequence of selected cores in detail. For this, we chose the Working Stage Analysis, which was developed to reconstruct the succession of different stages of preparation, production and reduction of single lithic tools and cores [16, 17]. Our analyses provide detailed descriptions of characteristic reduction sequences of blade and bladelet cores. Additionally, we present results of techno-typological investigations of lamellar and laminar components within AH IV in order to complement the investigations of the cores. For this study, 1024 artefacts, including all cores (n = 31), all formal tools (n = 277) and a sample of flakes, blades and bladelets (n = 685) were analyzed by a common metrical protocol and techno-typological attribute analysis (Table 1; Fig 4).

**Table 1:** Hohle Fels, AH IV. Analyzed artefacts.

Hohle Fels AH IV—studied artefacts	N	%
cores	31	3.03
unmodified blanks	685	66.89
tools	216	21.09
tools with core function	61	5.96
chunks	30	2.93
retoucher (quarcite)	1	0.10
total	1024	100

### 3.1. Attribute analysis

All artefacts were investigated according to a detailed techno-typological protocol. By this metrical data, typological and technological data of cores, blanks and tools, such as reduction angles, position and mode of modified edges, have been evaluated. Blank categories and types were estimated for all artefacts, including cores and tools. Moreover, raw material type and quality (micro-, fine- and coarse-grained) and, if possible, the raw material source were evaluated for every single artefact. Cores, blank and tools were described in detail according to technological and typological characteristics. Blanks were categorized according to the following criteria: flakes (>/ = 1 cm max. length), blades (double as long as wide, max. width >/ = 12 mm acc. to Tixier), bladelets (double as long as wide, max. width 7–11.99 mm), microblades (double as long as wide, < 7 mm) and bladelets from burin-cores = lamellar burin spalls /lbs; double as long as wide, max. width < 12 mm, usually two ventral faces and a triangular or trapezoidal cross-section) [18]. Further blanks including formal tools and cores on blanks were investigated according to the same techno-typological and metrical protocol. In this study, we compare results of the WSA with metrical and technological data of blades, bladelets, microblades and lamellar burin spalls such as butt type, bulb, lip, bulbar scar and blank profile.

**Fig 5:**
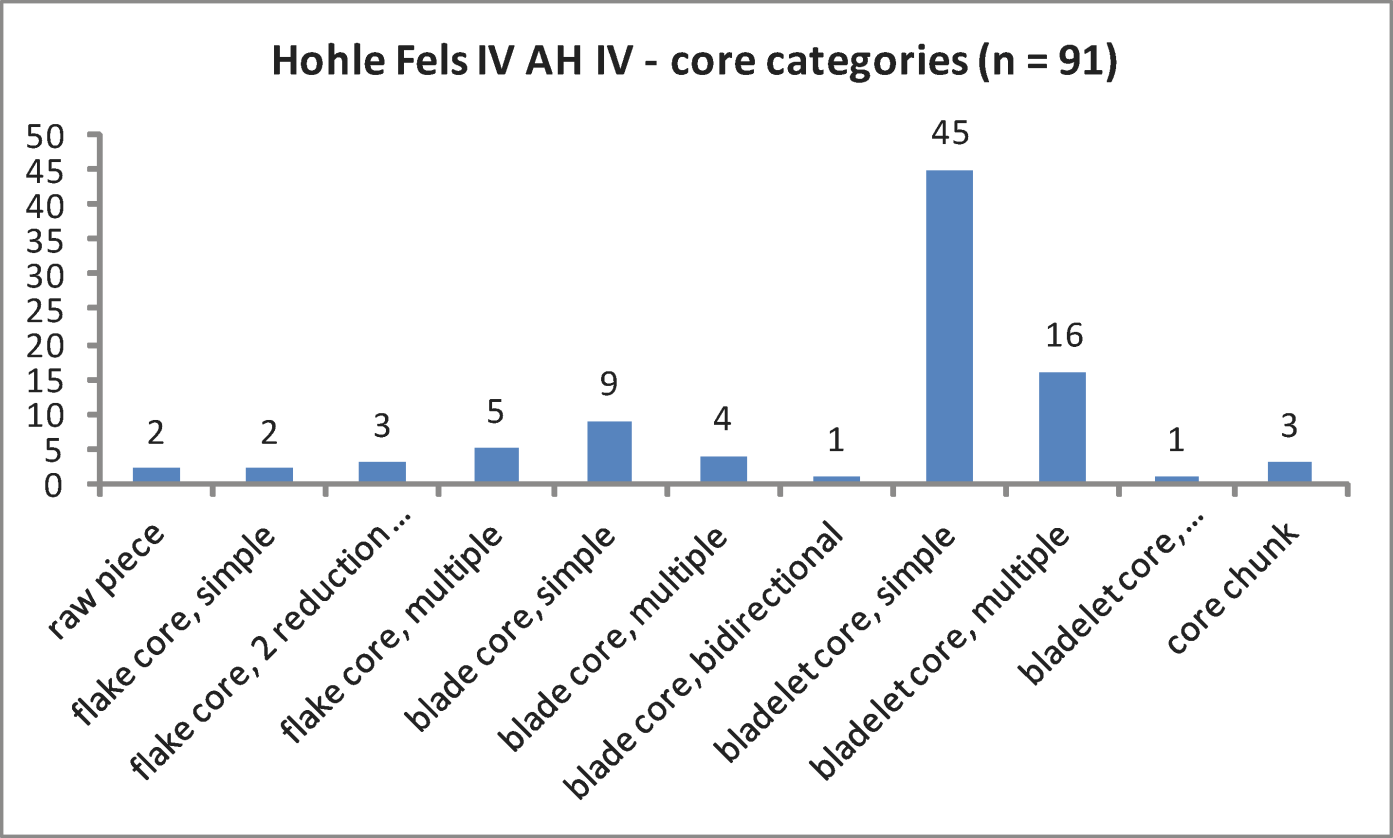
Hohle Fels, AH IV. Core categories including formal tools with core function.

**Fig 6:**
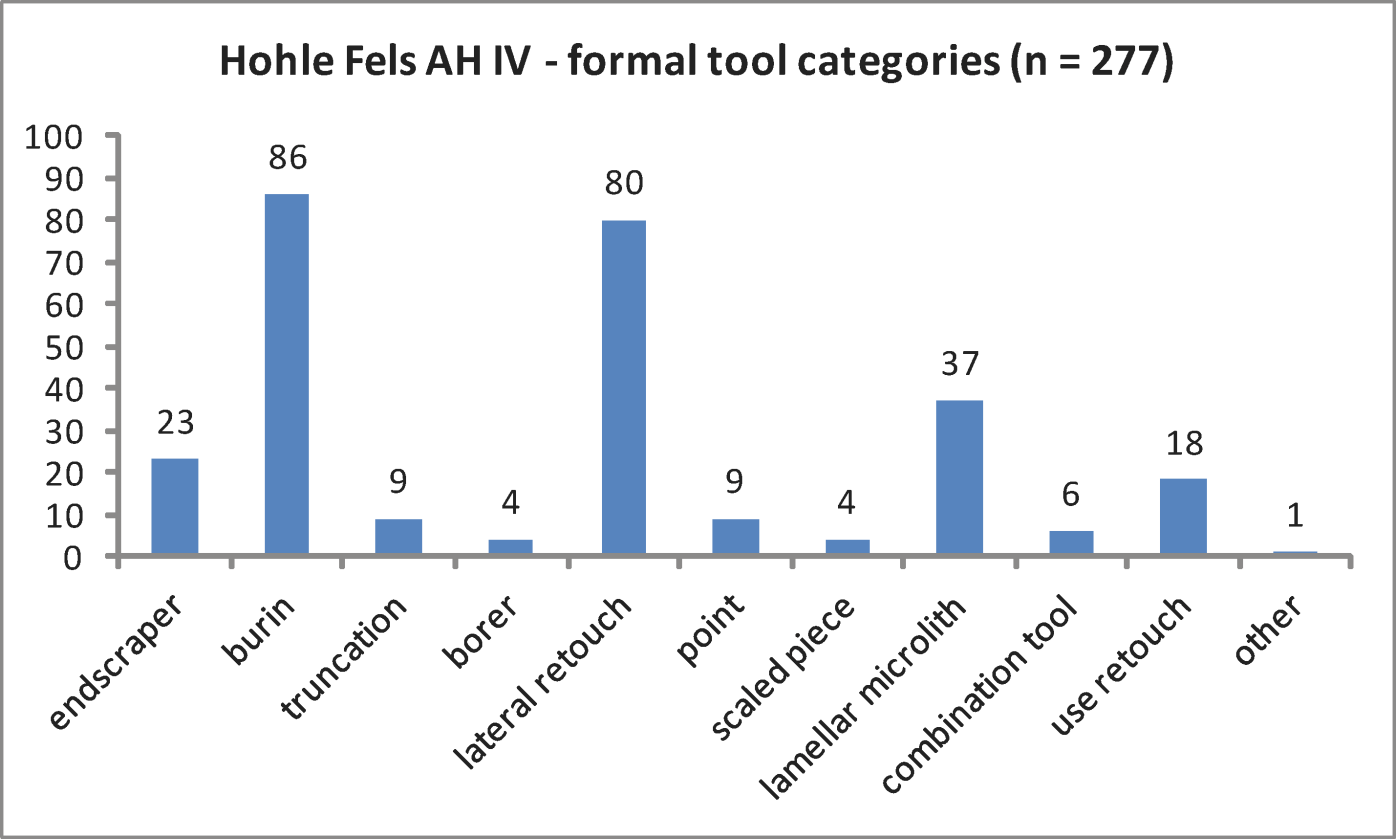
Hohle Fels AH IV. Formal tool categories including bladelet cores.

**Fig 7:**
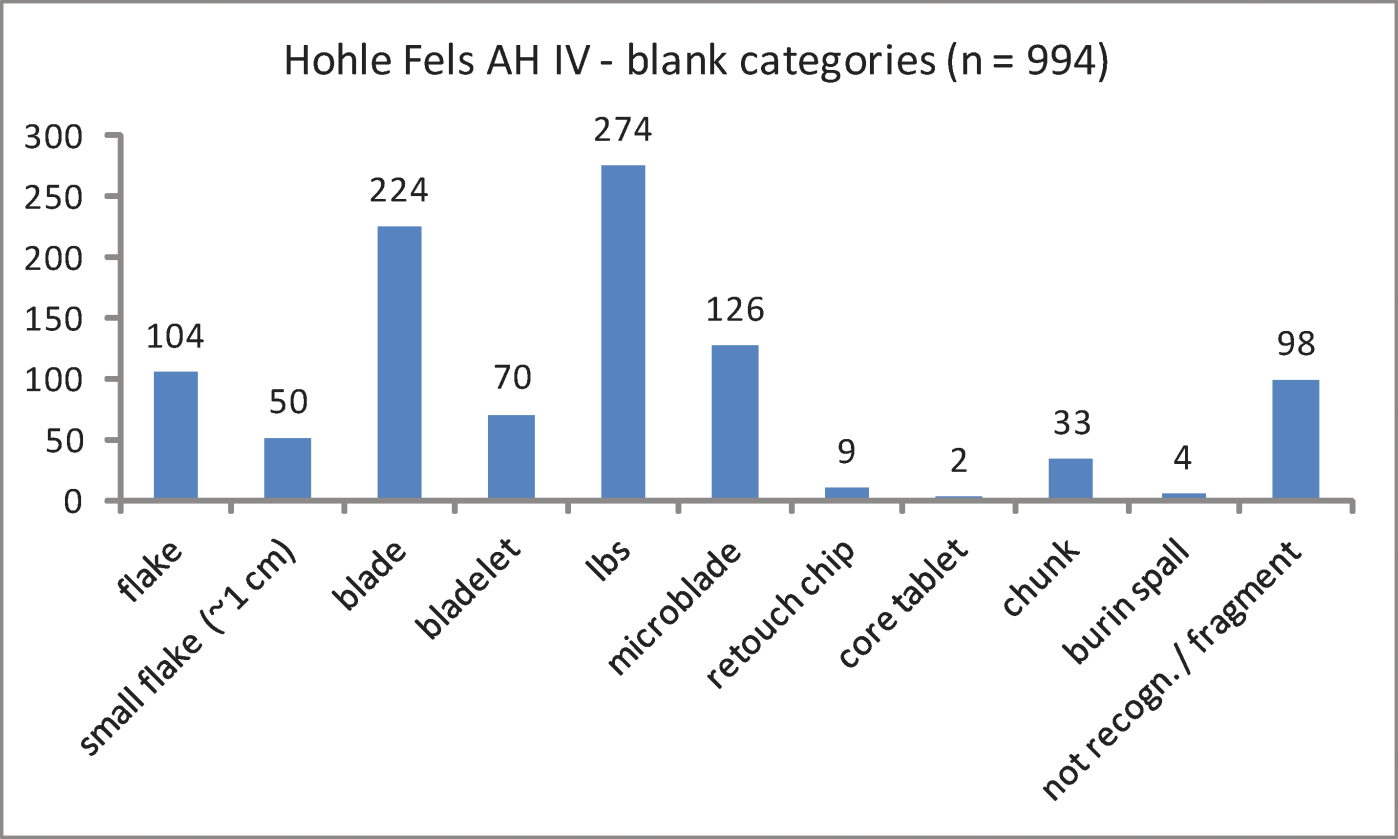
Hohle Fels, AH IV. Investigated blanks including modified pieces (tools and cores on blanks).

The regular cores exhibit two raw pieces, nine flake cores, ten blade cores and three core chunks (Table 2; Fig 5). All bladelet cores are formal tools (n = 52), such as carinated and nosed endscrapers as well as carinated and busked burins. Different burin types exhibiting multiple lamellar negatives are the dominating bladelet core category. The high share of burins in the tool assemblage reflects the importance of formal tools with bladelet core function (Table 3; Fig 6). Furthermore, we analyzed a sample of 685 unmodified blanks by a techno-typological attribute list. Additionally, we determined blank types and technological properties for all tools and cores by the same protocol. Among the investigated blanks, bladelets and microblades from burins = lamellar burin spalls; lbs) are most numerous (Table 4; Fig 7). Among the regular lamellar blanks, microblades are more numerous than bladelets.

**Table 2:** Hohle Fels, AH IV. Cores including formal tools with core function.

Hohle Fels AH IV—cores	core types	N	%	N	%
raw piece	raw piece	2	2.20	2	2.2
flake core	flake core, simple	2	2.20	10	10.99
	flake core, 2 reduction faces	3	3.30		
	flake core, multiple	5	5.49		
blade core, simple	sub-prismatic	6	6.59	8	8.79
	sub-pyramidal	1	1.10		
	sub-cylindric	1	1.10		
blade core, multiple	sub-cylindric	3	3.30	3	3.3
blade core, bidirectional	sub-prismatic	1	1.10	1	1.1
bladelet core, simple	carinated endscraper	3	3.30	50	54.95
	nosed endscraper (thick)	1	1.10		
	nosed endscraper (flat)	3	3.30		
	carinated burin	6	6.59		
	burin, multiple	3	3.30		
	burin, dihedral	6	6.59		
	burin on truncation*	25	27.47		
	burin on breakage	2	2.20		
	sub-cylindric	1	1.10		
bladelet core, multiple	sub-prismatic	1	1.10	13	14.29
	carinated endscraper	1	1.10		
	carinated burin	1	1.10		
	busked burin	1	1.10		
	burin, multiple	2	2.20		
	burin, dihedral	6	6.59		
	burin on truncation	1	1.10		
bladelet core, bidirectional	double burin, dihedral	1	1.10	1	1.1
core chunk		3	3.30	3	3.3
total		91	100	91	100

*One carinated burin on truncation.

**Table 3:** Hohle Fels AH IV. Formal tools including bladelet cores.

category	tool type	N	%
endscraper	simple	4	1.44
	simple, unilateral retouch	5	1.81
	simple, bilateral retouch	4	1.44
	simple, circulating retouch	3	1.08
	simple, use retouch	1	0.36
	simple / carinated	1	0.36
	carinated	1	0.36
	carinated, bilateral retouch	1	0.36
	nosed	1	0.36
	nosed, bilateral retouch	2	0.72
endscraper-burin	nosed endscraper / burin on truncation	2	0.72
	carinated endscraper / carinated burin	2	0.72
burin	simple	9	3.25
	simple; borer	1	0.36
	simple; truncation	2	0.72
	simple; unilateral retouch	2	0.72
	simple; unilateral retouch; notch	1	0.36
	simple; bilateral retouch	1	0.36
	simple; use / sediment	1	0.36
	carinated	6	2.17
	carinated; unilateral retouch	1	0.36
	carinated; dihedral	1	0.36
	carinated; on truncation	2	0.72
	busked; dihedral; unilateral retouch	1	0.36
	dihedral	18	6.50
	dihedral; splintered piece; unilateral retouch	1	0.36
	dihedral; notch	1	0.36
	dihedral; on truncation	1	0.36
	on truncation	27	9.75
	on truncation; truncation, oblique	2	0.72
	on truncation; unilateral retouch	2	0.72
	on truncation; bilateral retouch	1	0.36
	on truncation; Spitzklinge	2	0.72
	on truncation; use / sediment	1	0.36
	on breakage	1	0.36
	on breakage; unilateral retouch	1	0.36
borer	borer	3	1.08
	borer; bilateral retouch	1	0.36
truncation	truncation	6	2.17
	truncation, unilateral retouch	1	0.36
	truncation, bilateral retouch	1	0.36
	truncation, notch	1	0.36
lateral modification	unilateral	31	11.19
	bilateral	15	5.42
	denticulate	1	0.36
	notch	11	3.97
	unilateral; denticulate	1	0.36
	unilateral; notch	5	1.81
	unilateral; notch; use/sediment	1	0.36
	unilateral; use/sediment	12	4.33
	bilateral; denticulate	1	0.36
	sidescraper, double	1	0.36
	denticulate; use/sediment	1	0.36
point	point, bilateral retouch	1	0.36
	Spitzklinge	6	2.17
	Spitzklinge; use/sediment	2	0.72
lamellar microlith	bladelet, unilateral retouch	1	0.36
	bladelet, bilateral retouch; use retouch	1	0.36
	bladelet, use retouch	3	1.08
	microblade, unilateral retouch	1	0.36
	lbs, unilateral retouch	7	2.53
	lbs, unilateral retouch; use retouch	17	6.14
	lbs, use retouch	5	1.81
	borer on lbs,use retouch	1	0.36
	borer on microblade, use retouch	1	0.36
others	splintered piece	4	1.44
	unilateral retouch; splintered piece	1	0.36
	bilateral retouch; splintered piece	1	0.36
	use / sediment retouch	18	6.50
	retoucher with impact marks	1	0.36
total		277	100

**Table 4:** Hohle Fels, AH IV. Investigated blanks including modified pieces (tools and cores on blanks).

blanks	N	%	N	%
flake	104	10.46	154	15.49
small flake (~1 cm)	50	5.03		
blade	224	22.54	224	22.54
bladelet	70	7.04	470	47.29
lbs	274	27.57		
microblade	126	12.68		
retouch chip	9	0.90	146	14.68
core tablet	2	0.20		
chunk	33	3.32		
burin spall	4	0.40		
not recogn. / blank fragment	98	9.86		
Total	994	100	994	100

### 3.2. Working Stage Analysis

**Fig 8:**
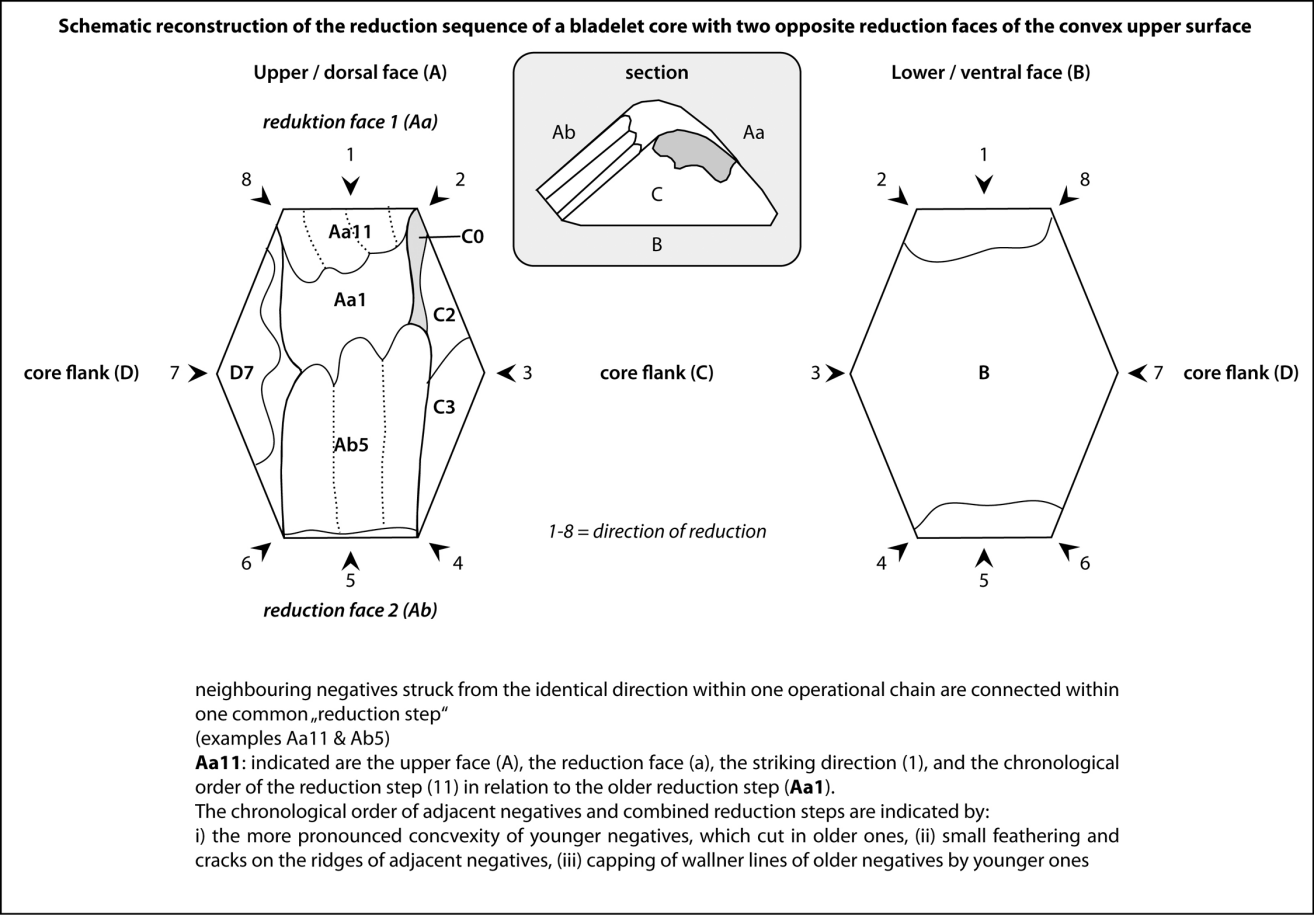
Working Stage Analysis. Explanation of the method.

The Working Stage Analysis = WSA), as developed [16, 17] for the reconstruction of production processes of bifacial tools and cores, was enhanced for the description of multi-platform cores by the authors [19, 20]. Neighboring negatives struck from the same direction within one operational step are subsumed under one "working stage" (Fig 8). Usually a working stage is the result of a specific aim, e.g., the preparation of the primary lateral convexity of a core. In other cases, unintentional negatives such as the natural fracture plain or a single hinge negative are regarded as working stages as well. In Fig 8 working stage Aa11 encodes the upper face (A), the reduction face (a), the striking direction (1), and the chronological order of the reduction stage (11) in relation to the older one (Aa1). Furthermore, the chronological order of adjacent negatives and combined reduction steps are indicated by: i) the more pronounced convexity of younger negatives, which cut in older ones, (ii) small feathering and cracks on the ridges of adjacent negatives, (iii) capping of *Wallner lines* of older negatives by younger ones.

Before presenting the results of the technological investigations, the term *core* should be described in more detail. The necessity to do this derives from the fact that Aurignacian nosed and carinated endscrapers as well as carinated and busked burins were originally defined as tools. Today, researchers agree that these artefacts were mainly used as cores for the production of lamellar blanks [21, 22, 23]. Nevertheless, the importance of burins with multiple lamellar negatives serving as cores is still underestimated [19].

Cores are defined in the following way. They can be prepared on a raw nodule, a chunk or on any desired blank suitable for the intended purpose of producing specific products. Usually we differentiate flake, blade and bladelet cores. Cores exhibiting especially slim lamellar negatives (max. width < 7 mm) could further be interpreted as microblade cores. Bladelet cores, which are the focus of this article, are often produced on diverging blank categories such as flakes, blades or chunks. Over the last decade the core character of formal tools, especially burins, from EUP assemblages has increasingly become the focus of study [21, 22]. Burin-cores were described from the Initial Upper Palaeolithic of the Altai Mountains [24], the Spitsynian EUP industry of Central Russia [19], the Aurignacian of Luxembourg and eastern France [25], the evolved Aurignacian of Northwestern Europe [26, 27, 28]. In general, a core regularly serves as a volume from which different kinds of blanks are struck. A core typically preserves at least three intentionally produced blank negatives. This restriction is important in the context of early Upper Palaeolithic bladelet production systems, especially when considering the traditional perception of bladelet cores as tools. In other cases, core candidates with less than three reduction negatives should share identical features with other cores of the same assemblage, such as reduction angle, the specific preparation of striking platform and reduction face. A core exhibits at least one striking platform and one or more reduction surface from which blanks are struck. In general, the angle between striking platform and reduction surface should be steeper than 90°; in Hohle Fels AH IV, it usually ranges between 30° and 75°. Blade and bladelet cores often exhibit steep reduction angles of less than 60°. In order to control the outline of the desired blanks, and to achieve the distal and lateral convexity of the core, it often exhibits prepared convexities of one or more lateral flanks and the terminal end in order to control lateral and distal shapes of the produced blanks. Additionally, the latter preparation step prevents the accidental striking of the plunging flakes.

Blade and bladelet cores in particular usually exhibit negatives, which indicate that blanks were struck in series from a single platform in the same direction (unidirectional-parallel and unidirectional-convergent method). Such cores might display two or more striking platforms and reduction surfaces, which are reduced alternatively or sequentially. Cores typically show clearly prepared striking platforms. In some cases, "truncations" should be considered as striking platforms rather than as active edges of tools. Admittedly, a secondary function of a prepared striking platform (e.g., an oblique or concave truncation) as active edge is possible. In this context, formal tools such as carinated endscrapers or carinated burins are interpreted as bladelet cores if they exhibit at least three lamellar negatives struck from a prepared platform [29–31]. Burins in the assemblage presented here exhibit less than three lamellar negatives but adhere to the definition cited above (identical configuration of striking platform, reduction surface and reduction angle). In this case they can also be viewed as likely candidates for initial bladelet cores.

## 4. Results

In the following, detailed analyses of blade cores are presented reflecting the technological variability of core exploitation of the AH IV assemblage (examples 1–4). This study is complemented by the detailed technological analysis of a flake core exhibiting identical technological characteristics (example 5). Furthermore, four additional multiple bladelet cores featuring different technological concepts were investigated (examples 6–9). In a second step, the results are compared with results of technological investigations of a representative sample of blades and bladelets.

In a second step, the results of the WSA are compared with and discussed in the context of attribute analyses of laminar and lamellar blank products.

### 4.1. Working Stage Analysis of blade cores

The bulk of recorded blade cores can be described as sub-volumetric single platform cores with wide or narrow-faced reduction surfaces, which were reduced in a unidirectional manner. These finds can feature sub-prismatic/ -cylindrical reduction faces with parallel scar patterns and, to a minor extent, sub–pyramidal ones with parallel or convergent scar patterns. Plain striking platforms are dominant. Examples 1 and 2 are typical sub-cylindrical cores with unidirectional-parallel negatives (Fig 9.1 and 9.2). There is, though, only one example of a narrow-faced blade core with a unidirectional-convergent scar-pattern (Fig 9.3).

**Fig 9:**
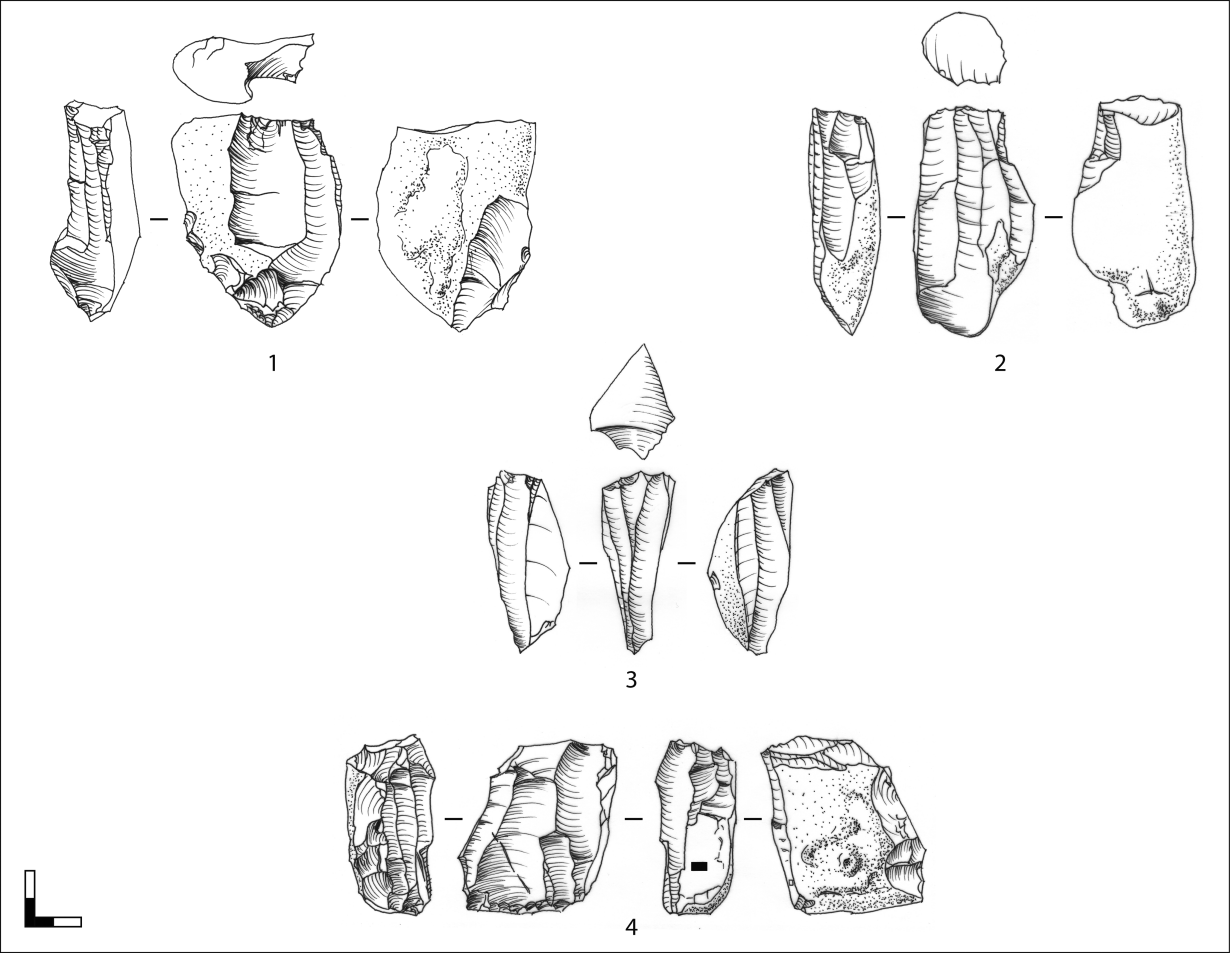
Hohle Fels, AH IV (GH 7). Blade cores. 1–2: unidirectional-parallel sub-prismatic blade cores; 3: unidirectional-convergent sub-cylindrical blade core; 4: opposed platform blade core, which was uni- and bidirectionally reduced from the prismatic main reduction face and the adjacent right lateral reduction face. Scale 1:2. Drawings: G. Bataille.

**Fig 10:**
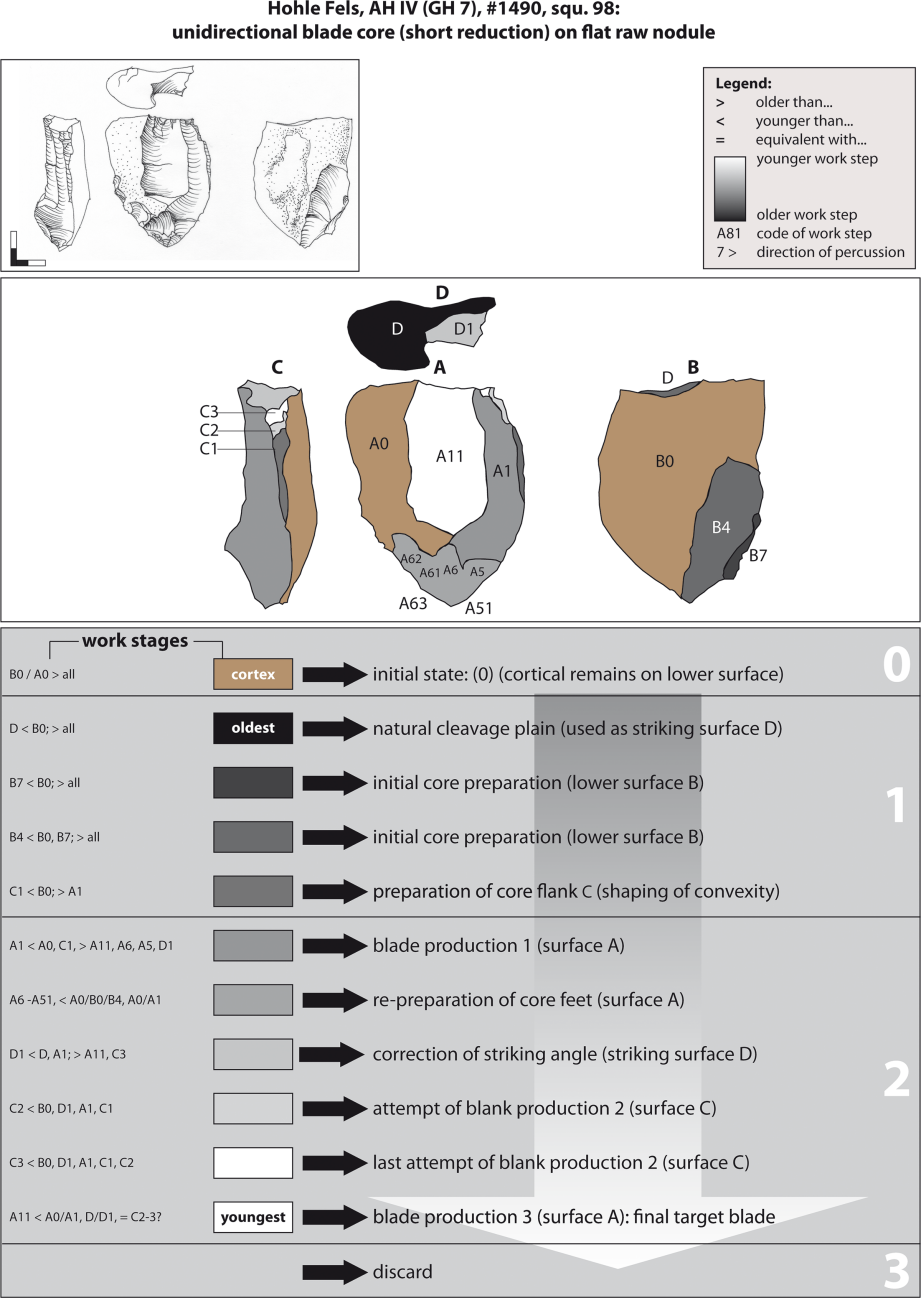
Hohle Fels, AH IV (GH 7). Working Stage Analysis. Example 1. Unidirectional-parallel blade core. Drawings: G. Bataille.

Example 1 represents a short unidirectional-parallel blade production sequence (Fig 10). A flat nodule from local Jurassic chert (stage 0 = initial state) was initialized on the lower surface B. Subsequently, the left core flank was prepared by longitudinal blows (stage 1 = core preparation). From the upper surface A, blades were struck in a unidirectional-parallel manner (stage 2 = blank production). The natural breakage plain D was thereby used as a striking platform. After correcting the striking angle by detaching a small flake from striking surface D (working stage D1), further blades were detached from the lateral supplementary face C (WS C2 & C3). Finally, at least one further blade was detached from the wide reduction surface A before the exploited core was abandoned (WS A11).

**Fig 11:**
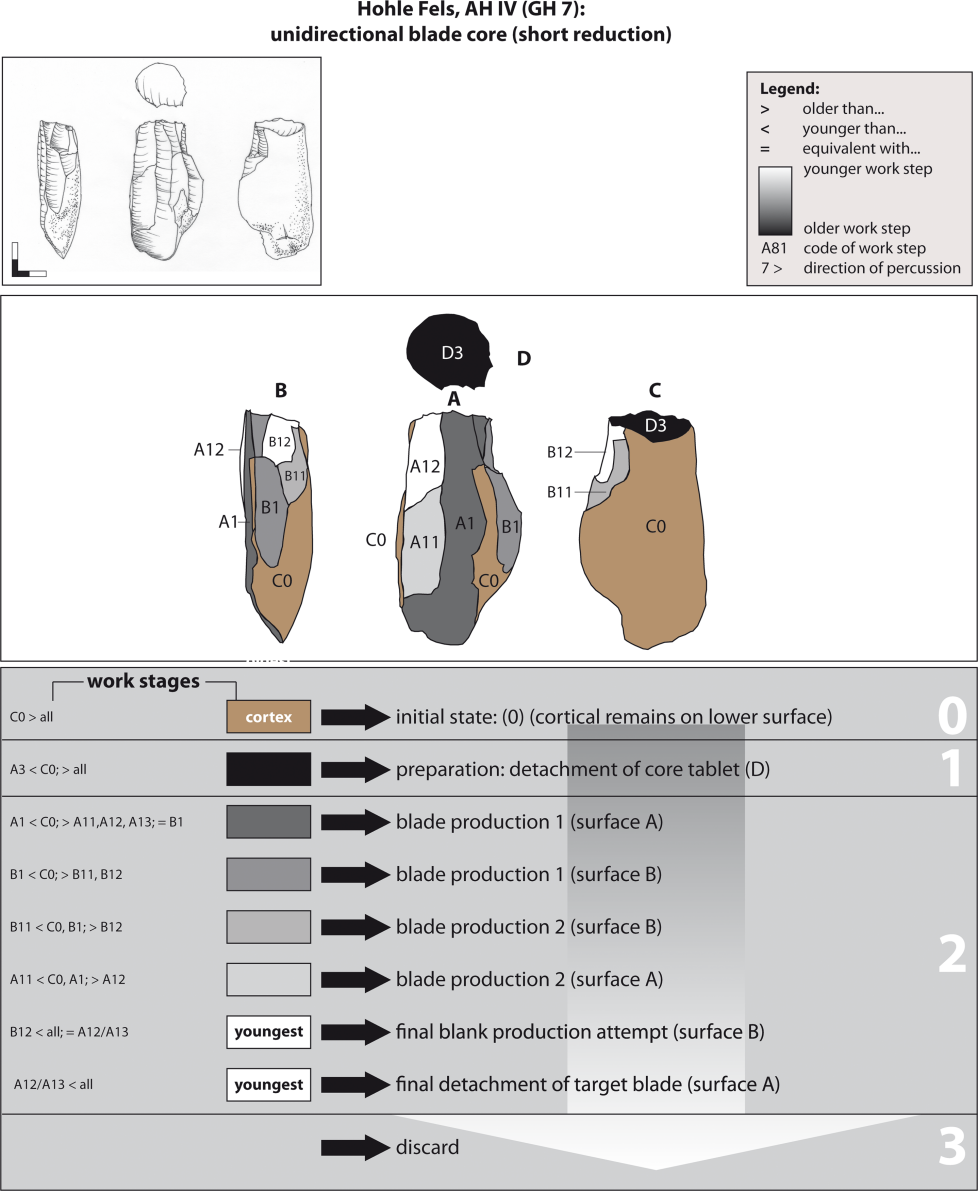
Hohle Fels, AH IV (GH 7). Working Stage Analysis. Example 2. Unidirectional-parallel blade core. Drawings: G. Bataille.

Example 2 represents a typical unidirectional reduction sequence from two adjacent reduction faces (A & B) (Fig 11). Similar to example 1, this core was initialized on a flat Jurassic chert (stage 0) at the upper surface A and the adjacent right flank (stage 1). Moreover, in order to initialize the core plunge of surface A, the lower surface B was primarily prepared in its lower lateral part (stage 1). The blank production stage 1 is represented by the alternating unidirectional-parallel flaking of the reduction surfaces A and B. The plain face D served as a flat striking surface, which has been formed by the detachment of a thick flake prior to the preparation of surfaces A and B. The negative of a wide *outrepassé* target blank represents WS A1. Again, a more or less exploited core was then discarded (stage 3).

**Fig 12:**
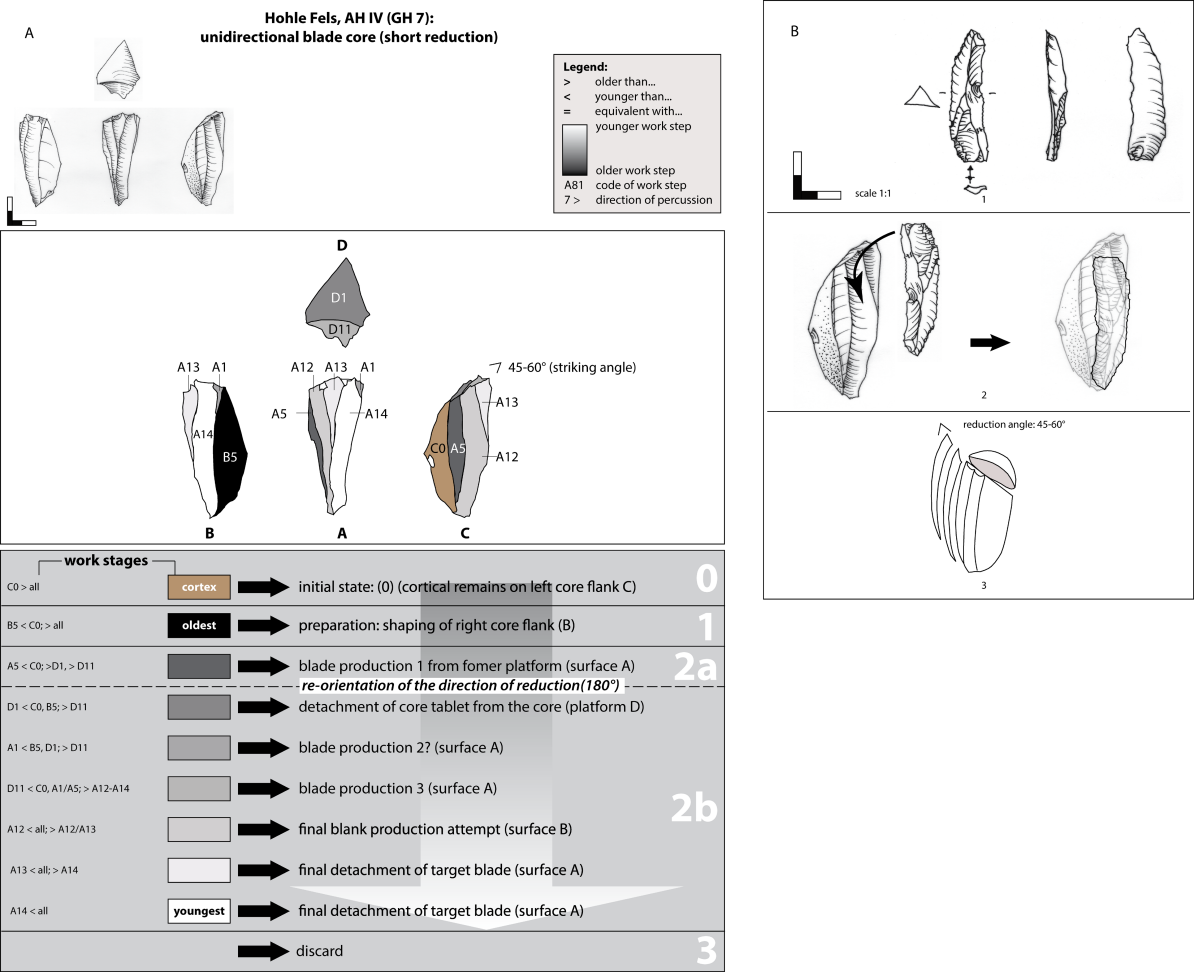
Hohle Fels, AH IV (GH 7). (A) Working Stage Analysis. Example 3. Unidirectional-convergent blade core. (B) Raw material unit of the unidirectional-convergent blade core and a blade with convergent outline, which fits on one negative. Drawings: G. Bataille.

A unidirectional-convergent blade reduction (example 3; Fig 12A) is indicated by only one example. Due to the shape of the lateral cortical remains on face C, the core was prepared on the fragment of a flat tabular Jurassic chert (stage 0). From the preparation phase 1, which was conducted from the opposite edge of the present striking platform, the lateral preparation of the right core flank B is conserved (WS B5). A core tablet (WS D1) was then detached, which formed a reduction angle of about 45–60° degrees. Afterwards, the knapper struck blades from reduction face D1 in a unidirectional-convergent manner (stage 2). The two slim negatives of WS A1 potentially indicate a short sequence of unidirectional bladelet (max. width 7–11.99 mm) and microblade (max. width <7 mm) production from the identical platform. Working stage A5 was struck from the end opposite the striking surface D1/D11, potentially from the identical striking platform WS B5. The distal end of negative A5 is capped by the striking platform negative D1, and represents an earlier phase of reduction. Embedded within the blade production sequence is the correction of the angle of the flat striking surface by the detachment of a further flake (WS D11). The small core was discarded in a highly reduced state.

The fact that this core was repeatedly rejuvenated is indicated by a slim laterally retouched blade with a triangular cross-section and convergent outline. Together with the core it forms a common work piece, as described by Weissmüller [32]. This piece derives from a reduction sequence prior to the application of striking surface D1 (Fig 12B). A section of its distal end fits to the negative of WS A12. Consequently, prior to the production of striking surface D1, which exhibits a steep reduction-angle of 45–60°, the knapper made another reduction face. The angle is consistent with that of the retouched blade, indicating a core configuration and blade exploitation intended from the beginning of the knapping process and maintained over the course of at least two rejuvenation phases that are connected with recurrent detachments of core tablets (WS D1 & D11). Moreover, the flat striking surface of the blade might point to at least one earlier stage of striking platform preparation. To conclude, the blade production phase indicated in the refitting can be described as a working stage in between the recognized preparation of the right core flank (WS B5, phase 1) and the detachment of the core tablet (WS D1) during blank production phase 2 (Fig 12A). This particular convergent blade/bladelet core exhibits unidirectional convergent reduction stages for blade production that were conducted from two opposed platforms within two succeeding main phases of core preparation and exploitation. Main phase 1 is indicated in working stage A5 and B5, and main phase 2 in the remaining working stages.

**Fig 13:**
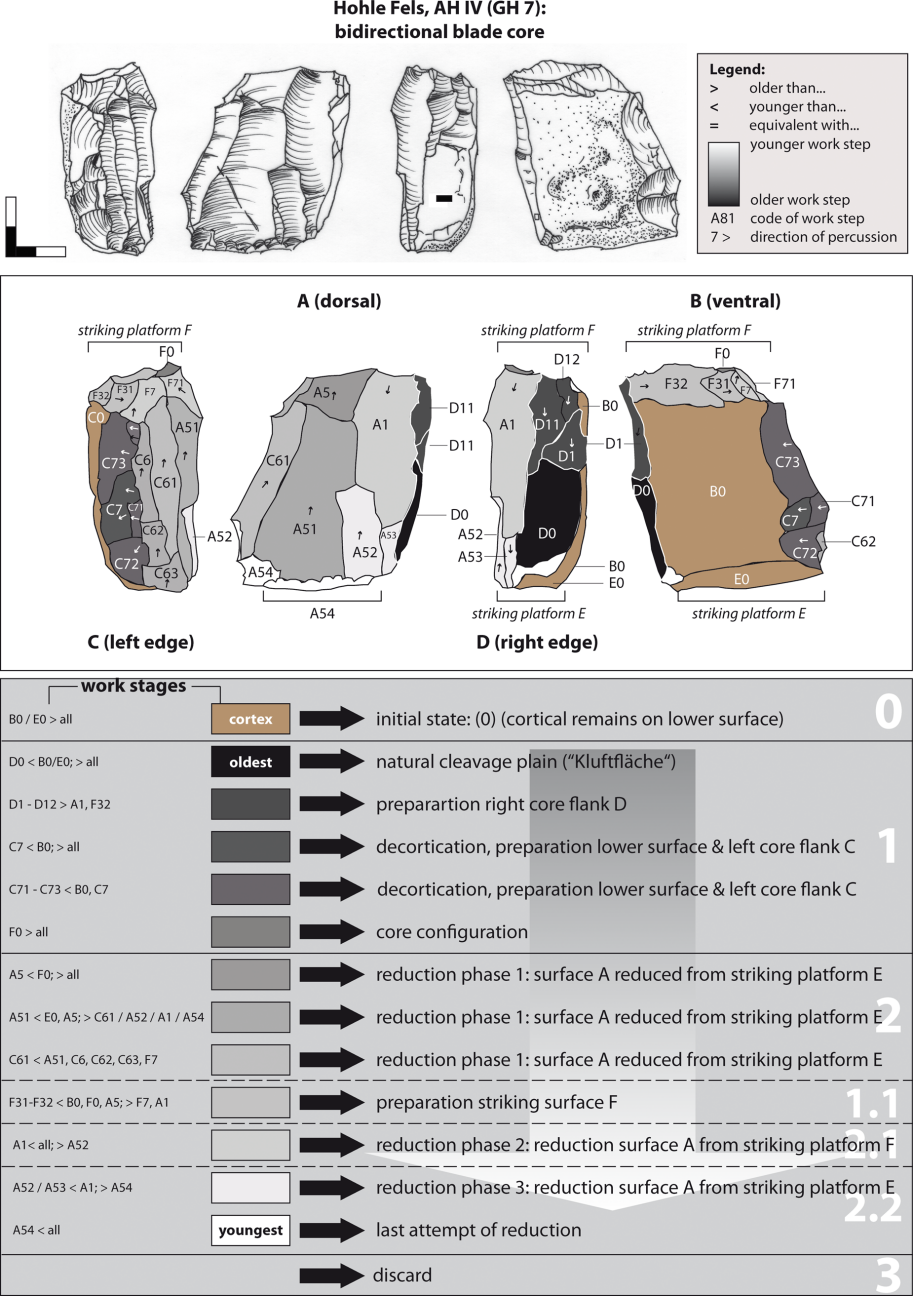
Hohle Fels, AH IV (GH 7). Working Stage Analysis. Example 4. Opposed-platform blade core. Drawings: G. Bataille.

A comparatively complex reduction history, consisting of successive unidirectional sequences from two opposed striking platforms and a bidirectional-parallel sequence of blade production, is represented by example 4 (Fig 13). Again, a flat nodule from a grey Jurassic chert was initialized through lateral preparation and the subsequent formatting of one main reduction face (A), with the lower surface remaining cortical. The lower cortical edge and the opposed edge were used as the striking platform, which was formed by unidirectional parallel negatives.

**Fig 14:**
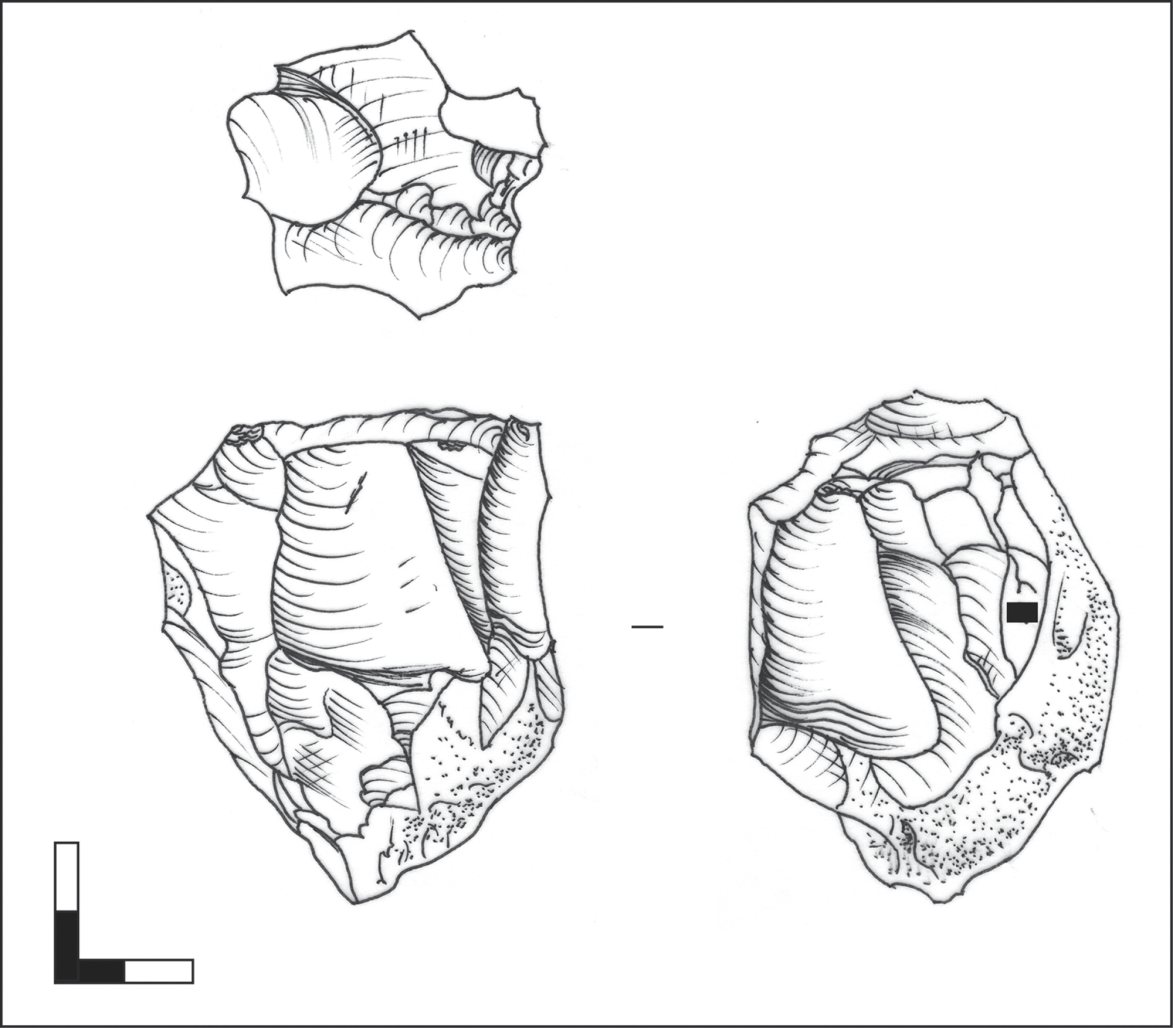
Hohle Fels, AH IV (GH 7). Example 5. Unidirectional-parallel flake core. The morphological features of the reduction face and of the carefully prepared flat striking platform are identical to the typical unidirectional-parallel sub-prismatic blade cores of the assemblage. Scale 1:1. Drawings: G. Bataille.

A flake core (example 5) is best described in the context of the above-mentioned blade cores since they share technological and formal similarities (Fig 14). This unidirectional prismatic flake core was mainly exploited from one striking platform carefully prepared by laminar and non-laminar negatives. The core structure is identical to that of regular sub-prismatic blade cores. Obviously, the main striking platform was produced by the detachment of core tablets and bigger flakes. Longitudinal flakes were produced by unidirectional-parallel series from the sub-prismatic reduction surface. The striking angle was adjusted repeatedly by longitudinal negatives.

In summary, the method used to produce blades is consistent within the assemblage, which exhibits sub-volumetric reduction surfaces that are usually reduced from plain striking platforms in a unidirectional manner. Bidirectional exploitation strategies are clearly underrepresented. The preparation of blade cores usually began with the preparation of a plain striking platform and a reduction angle of around 60°. Distal convexity was achieved by a number of parallel or convergent negatives. The same is true within Geißenklösterle AH II and III [33], where lateral preparation was not extensive. In some cases parallel negatives struck orthogonally to the reduction direction formed a core crest along one side. The reduction sequence was indicated by the detachment of such a prepared crest or by the use of natural ridges (“Leitgrat” according to Hahn [33]). Real flake cores are configurated and reduced in an identical manner and might be former blade cores.

#### 4.1.1. Blade production

**Fig 15:**
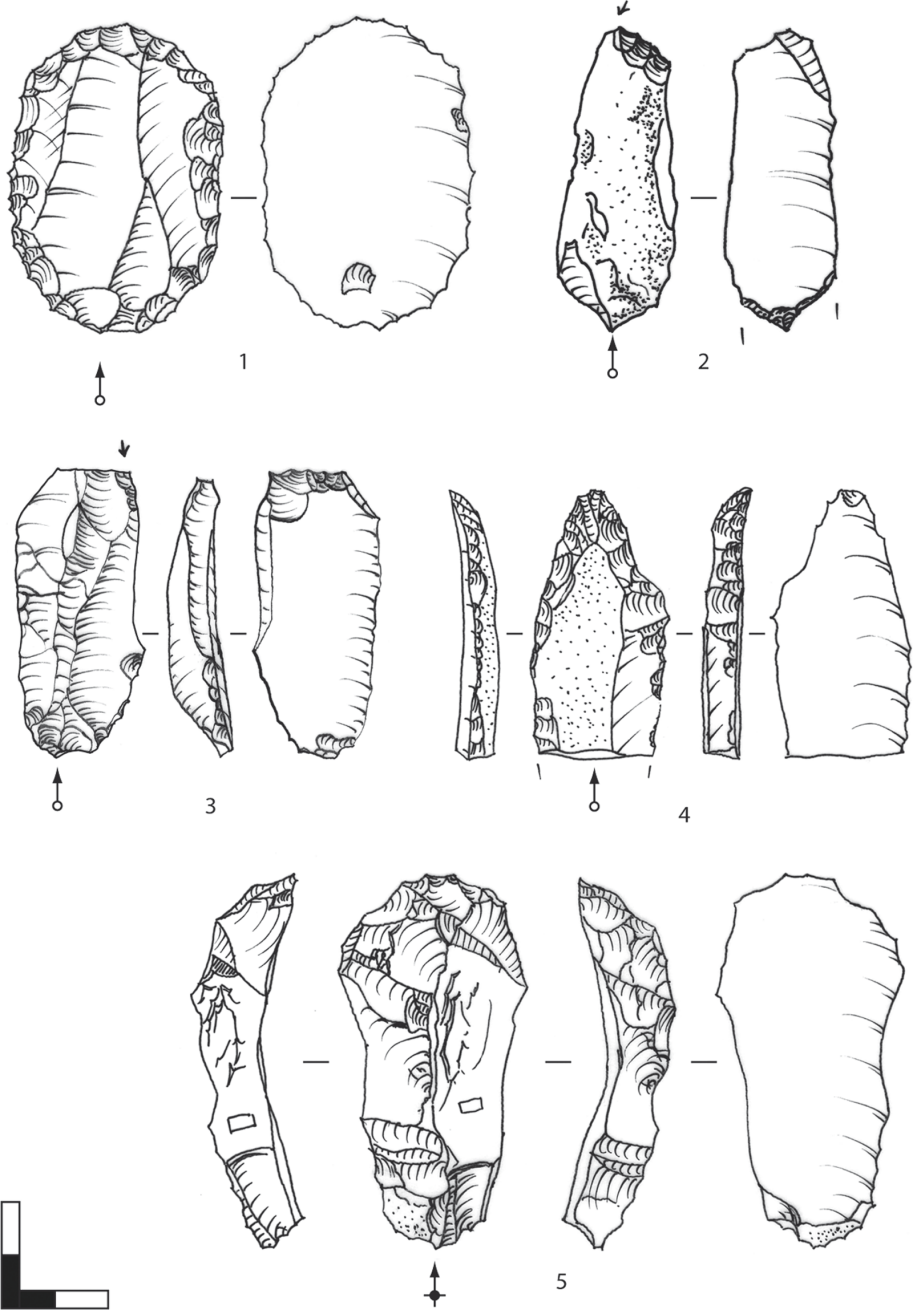
Hohle Fels Cave, AH IV (GH 7). Target products of blade/flake production. Simple tools. 1: flake with stepped retouch; 2: simple burin on blade; 3: burin on truncation; 4: flat nosed endscraper with bilateral retouch & lamellar negatives; 5: carinated endscraper. Scale 1:1. Drawings: G. Bataille.

**Fig 16:**
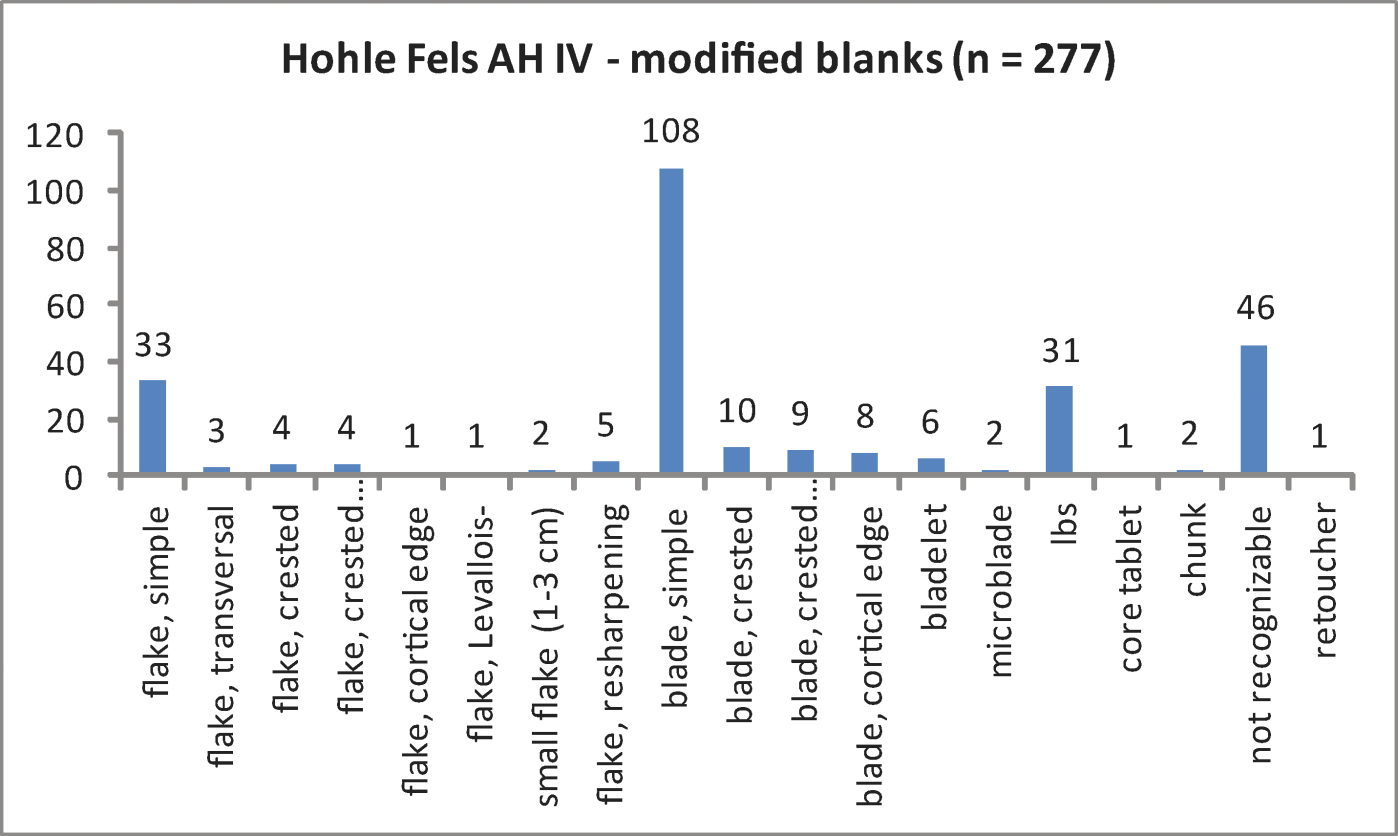
Modified blanks.

**Fig 17:**
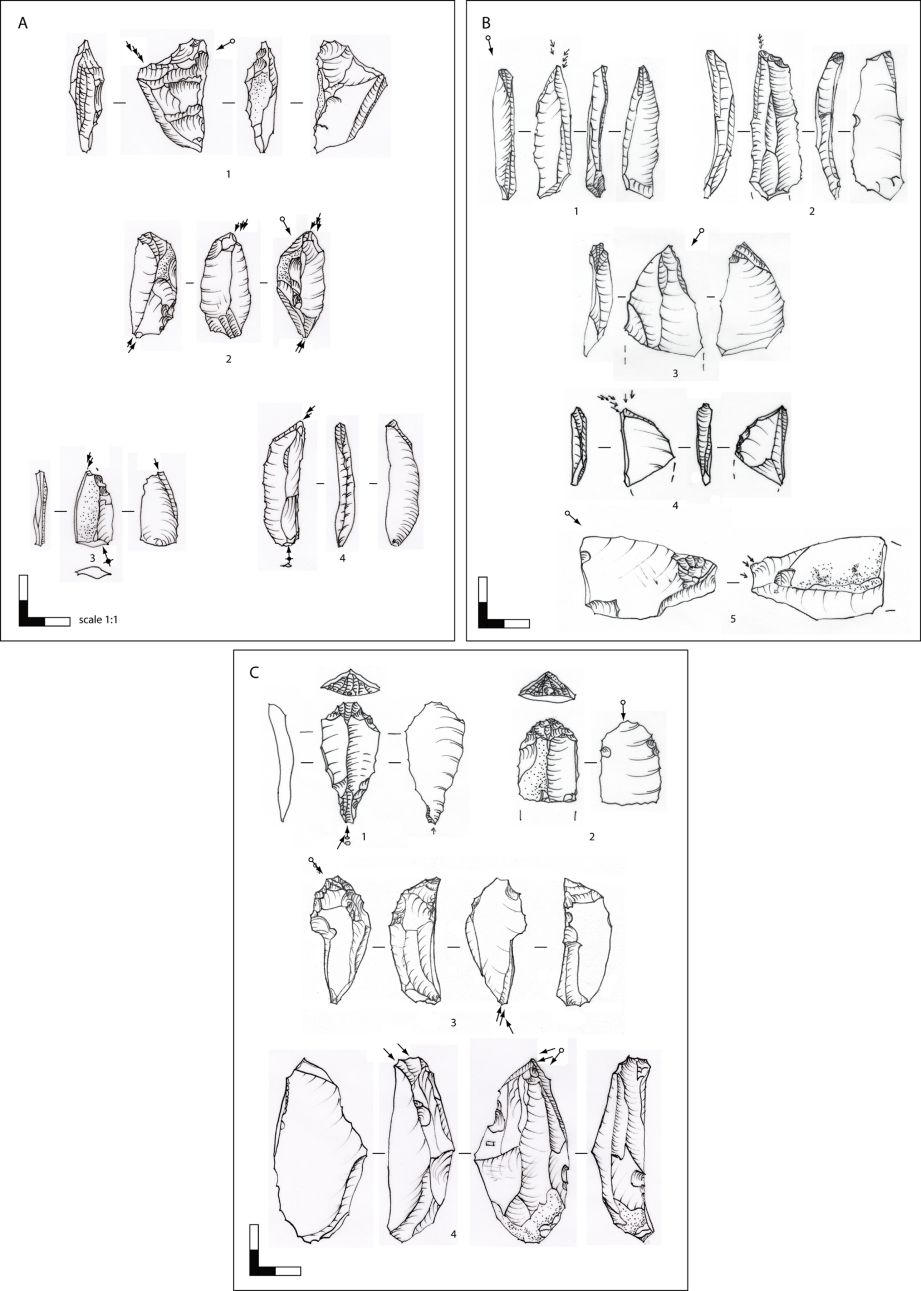
Hohle Fels, AH IV (GH 7). Target products of blade production. Burin cores on blades and flakes. (A) 1: burin on truncation/ unidirectional bladelet core; 2: opposed platform unidirectional microblade core; 3: fragment of a multiple burin/ microblade core; 4: oblique burin/ microblade core. **(B)** 1: dihedral burin; 2: burin on truncation; 3: carinated burin; 4: busked burin; 5: flake with multiple lamellar burin blows. (C) 1–2: carinated/ nosed endscrapers on blades with small reduction faces for microblade production; 3: combined carinated endscrapers (proximal end) for the production of small curved/twisted microblades and dihedral burin (terminal end) for the production of long straight and on-axis twisted baldelets/microblades; 4: busked burin. Scale 1:1. Drawings: G. Bataille.

The blade production strategy of AH IV was aimed toward comparatively thick and short blanks for further modification. Blades in AH IV usually exhibit straight or slightly curved profiles. The assemblage includes the well-known Aurignacian tool-set of lateral retouched blades and, in some cases, reworked pieces with stepped retouch ("Aurignacian retouch") as well as pointed blades with and without stepped retouch, endscrapers and simple burins (with fewer than 3 lamellar negatives) (Fig 15). Laterally retouched blades are most numerous. Endscrapers and burins were more often produced on blades than on flakes (Fig 16; Table 5). An important feature of this assemblage is the formal category of burins with multiple lamellar negatives (Fig 17A–17C). Such cores indicate the presence of a specific kind of bladelet/microblade production.

**Table 5:** Modified blanks. Lbs = lamellar burin spalls.

modified blanks	N	%	N	%
flake, simple	33	11.91	53	19.13
flake, transversal	3	1.08		
flake, crested	4	1.44		
flake, crested remnant	4	1.44		
flake, cortical edge	1	0.36		
flake, Levallois-	1	0.36		
small flake (1–3 cm)	2	0.72		
flake, resharpening	5	1.81		
blade, simple	108	38.99	135	48.74
blade, crested	10	3.61		
blade, crested remnant	9	3.25		
blade, cortical edge	8	2.89		
Bladelet	6	2.17	39	14.08
Microblade	2	0.72		
Lbs	31	11.19		
core tablet	1	0.36	50	18.05
Chunk	2	0.72		
not recognizable / fragment	46	16.61		
Retoucher	1	0.36		
Total	277	100	277	100

### 4.2. Working Stage Analysis of bladelet cores

**Fig 18:**
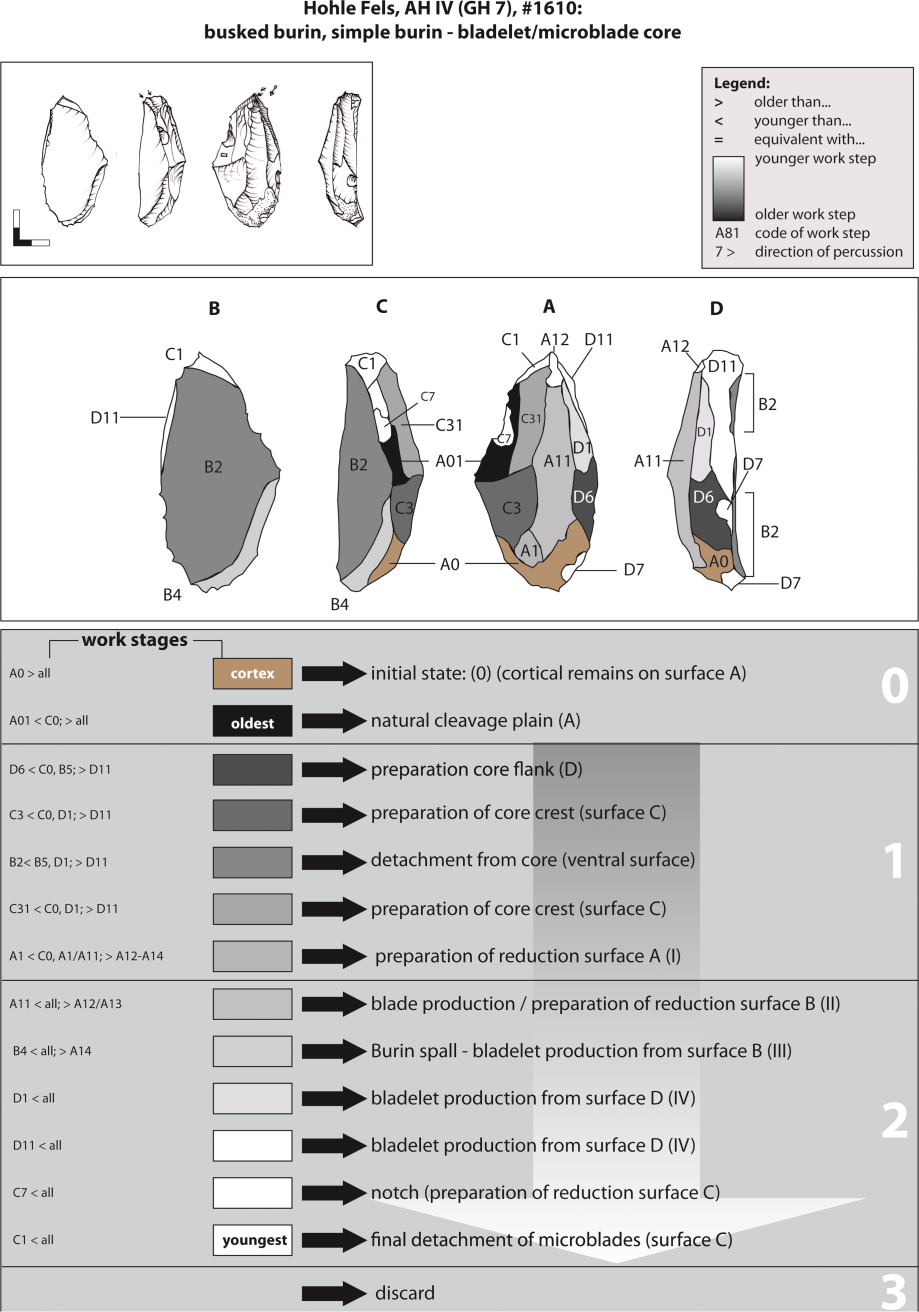
Hohle Fels, AH IV (GH 7). Working Stage Analysis. Example 6. Busked burin: bladelet core.

Example 6 illustrates a combined busked and simple burin on a thick primary flake (Fig 18). Cortical remains suggest that the selected raw piece was a nodule of grey Jurassic chert (stage 0). The oldest recognizable negative is a natural cleavage plain. First, lateral blows (WS D6; stage 1) formed the right core flank D. Later on, the left core crest C was formed (WS C3/C31). The next recognizable step conserves the detachment of the flake from the core (ventral surface B2). The laminar negative of WS A1 indicates an earlier sequence of blade production or, more likely, the formatting of surface A by laminar blows.

One single lamellar scar along the edge of surface B (WS B4) indicates blank production stage 1. The main, and potentially singular, bladelet/microblade sequence was applied on surface D from which bladelets/microblades were recurrently struck ("carinated burin"). WS D11 is also the striking surface for reducing the reduction face C1, which together with notch WS C7 characterizes the artefact as a busked burin. Interestingly, the preparation of the notch at the left lateral edge (WS C7), which functions as a border for delimiting the microblade reduction surface C, was not reached by the burin blows of WS C1. The detachment of bladelets from surface C (WS C1) indicates the last production step. Moreover, small splintering and negatives at the edge of the striking platform for reduction face C might point towards secondary use of the edge. The preparation and reduction of the busked / dihedral burin section (WS C1, D1 & D11) exhibits technological analogies with busked burins from the Aurignacian sites of Maisières-Canal and Trou du Renard (Belgium) [20, 27].

**Fig 19:**
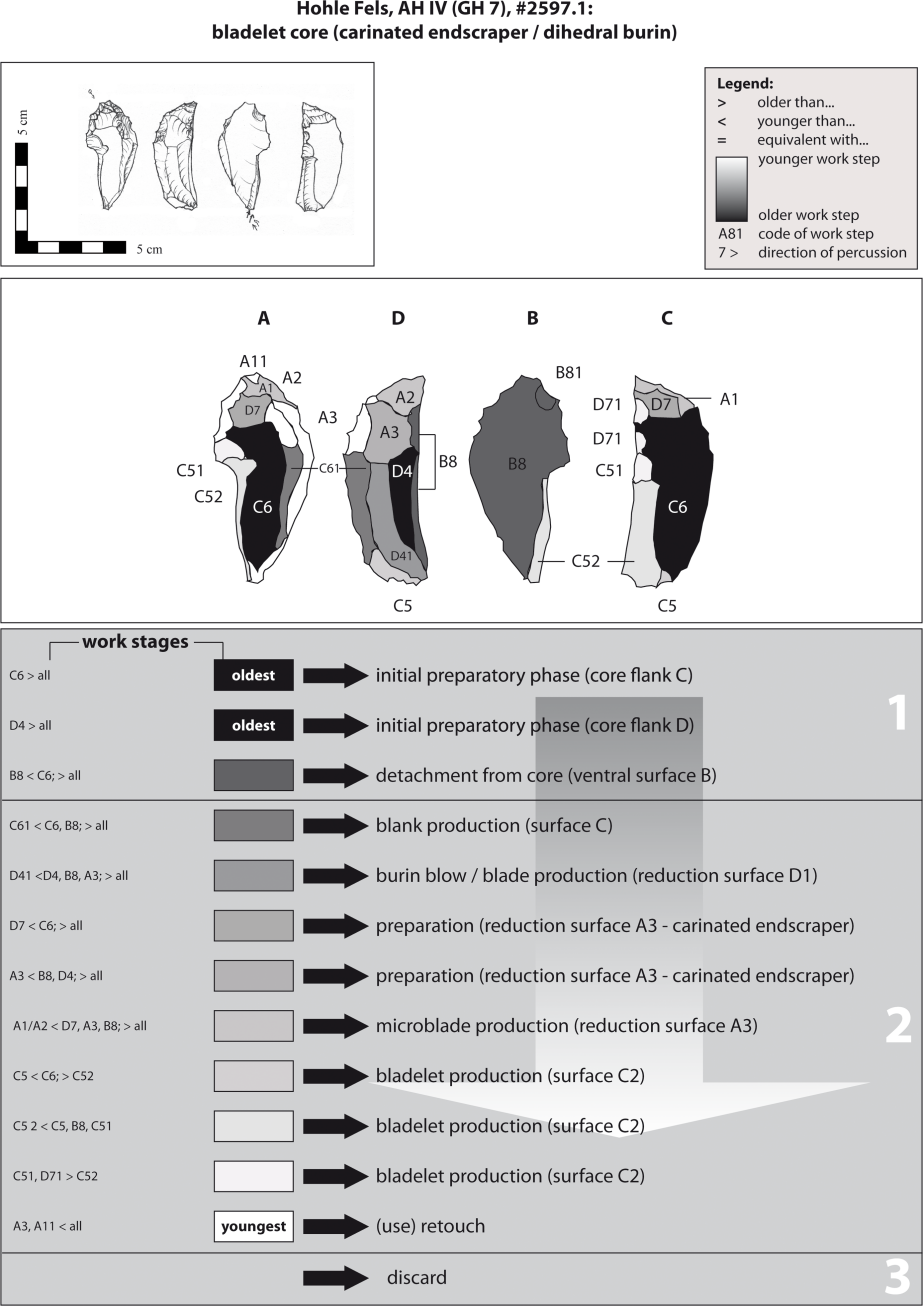
Hohle Fels, AH IV (GH 7). Working Stage Analysis. Example 7. Carinated endscraper / dihedral burin: opposed platform bladelet core.

From a typological point of view, example 7 is a combined tool (Fig 19). The knapper produced a carinated endscraper on the basal end and a dihedral burin on the terminal end of a thick longitudinal flake. From a technological point of view, the piece represents a multiple bladelet/microblade core used for the production of lamellar blanks from two opposed edges by the application of two different technological concepts. Knappers struck long and straight, on-axis twisted bladelets ("Dufour sub-type") from the proximal end, while detaching short curved and off-axis twisted microblades ("Roc-de-Combe sub-type") from the terminal end.

An early preparation stage of the core (WS D4) is indicated by negatives located on the right edge (D) of the piece. At the opposite face C, the negative C6 indicates the preparation of the left core flank. The blank was likely detached from the original core in a subsequent step (ventral surface B8). The bladelet production commenced from the proximal end. Working stage C61 represents the recognizable step of bladelet production. The negative runs along the middle ridge of the core. Younger negatives of lamellar production and preparatory negatives of reduction surface A cap this ridge. A younger sequence in the production of long bladelets through reducing the dihedral burin end is indicated in working stages D41 and D4. Subsequently, the proximal end was reduced along the reduction faces C and D by further dihedral burin blows (WS C5, C51 & C52). Moreover, working stage C63 attests to an intermediate preparation of the striking platform for the bladelet production sequence of working stage C52. Afterwards, reduction surface 3 (carinated endscraper) was prepared at the terminal end (e.g., WS D7/A3). In contrast to the dihedral burin end, the knapper struck small bladelets/microblades with curved and off-axis twisted profiles from the opposite edge. Ventral face B functioned as a striking platform (WS B8 & B81). After a final use of the active endscraper edge (WS A3), the piece was discarded.

**Fig 20:**
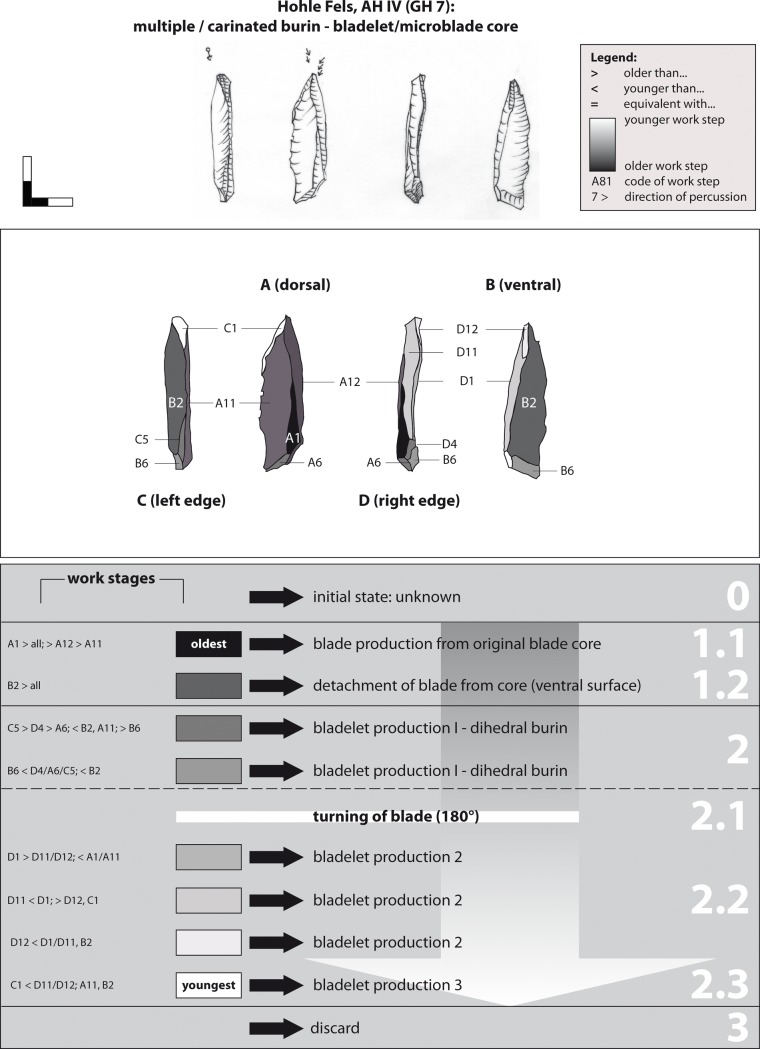
Hohle Fels, AH IV (GH 7). Working Stage Analysis. Example 8. Carinated/ multiple burin on blade: bladelet core.

A multiple burin-core on a blade exemplifies a typical sequence of microblade production (example 8; Fig 20). Although the piece represents a small artefact, its production and reduction history is complex. The core was produced on grey Jurassic chert. The original state of the raw piece (stage 0), meaning whether it was a round or flat nodule or plaquette, cannot be evaluated due to the lack of cortical remains. The earliest negatives (WS A1) derive from a former sequence of blade production (stage 1) prior to the detachment of the blank from the core (WS B2). This blank was subsequently prepared and reduced as a burin-core. Microblades (stage 2) were produced in the course of three subsequent reduction steps. First (stage 2.1), at least four microblades were produced from the basal edge of the blade (WS C5, D4, A6 & B6). At this stage of exploitation, the piece can formally be classified as dihedral burin. As a next step, the blade and the striking direction were re-oriented (180°) in order to produce bladelets and microblades from the opposite distal end of the core. In the course of the second production sequence (stage 2.2), comparatively long bladelets/ microblades were struck from the right lateral edge of the core (WS D1, D11 & D12). In a last step, the wide bladelet negative D11 functioned as the striking platform of reduction face C1. Knappers produced small and slim microblades from this small reduction face at the distal end of the left lateral edge. Afterwards, the double dihedral burin was discarded.

**Fig 21:**
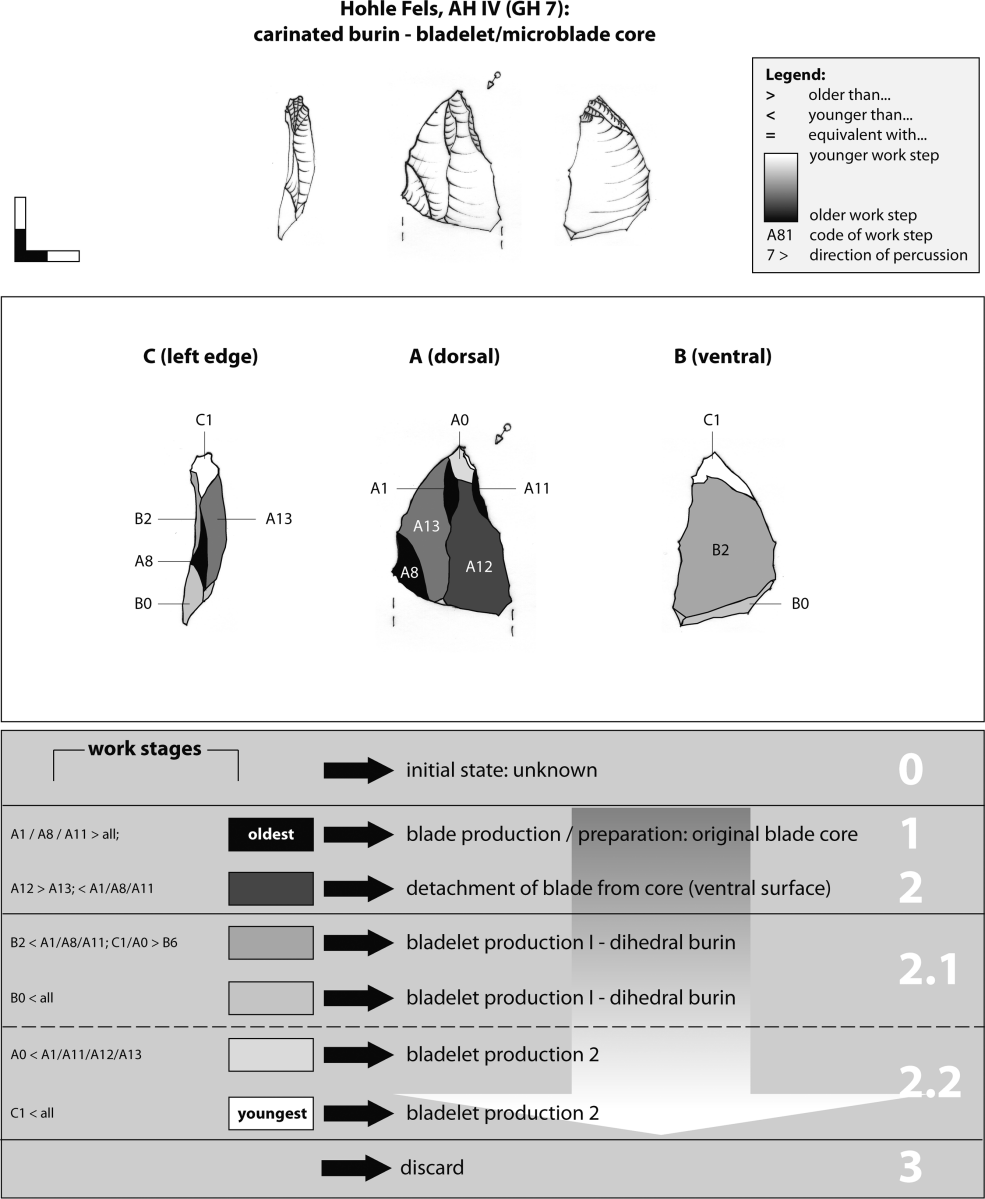
Hohle Fels, AH IV (GH 7). Working Stage Analysis. Example 9. Carinated burin on flake: bladelet core.

Example 9 demonstrates the reduction of a carinated burin on a flake (Fig 21). Due to the lack of cortical remains, the initial state of the raw piece is again unknown. The bladelet core was produced on brown Bohnerz chert (stage 0). Large negatives on dorsal face A indicate the preparatory and/or blank production phase of the original nodule (stage 1). In a next step, a longitudinal blank was detached from the original (WS A12). Finally, the blank was detached from the core, as indicated on ventral face B2 (stage 2.1). This blank was used to prepare and reduce a burin-core. The knapper established the truncated-like striking platform at the proximal end (WS A0) as a preparatory step for the exploitation of the reduction surface (stage 2.2). Afterwards, target blanks were struck along the proximal left lateral edge of the blank in a unidirectional manner (WS C1). It remains unclear if the medial breakage of the piece occurred prior to or after applying lamellar reduction surface C1. It is possible that the burin edge was used as an active edge after the microblade production phase, with the support breaking in the course of other related activities.

#### 4.2.1. Bladelet production. Target products

**Fig 22:**
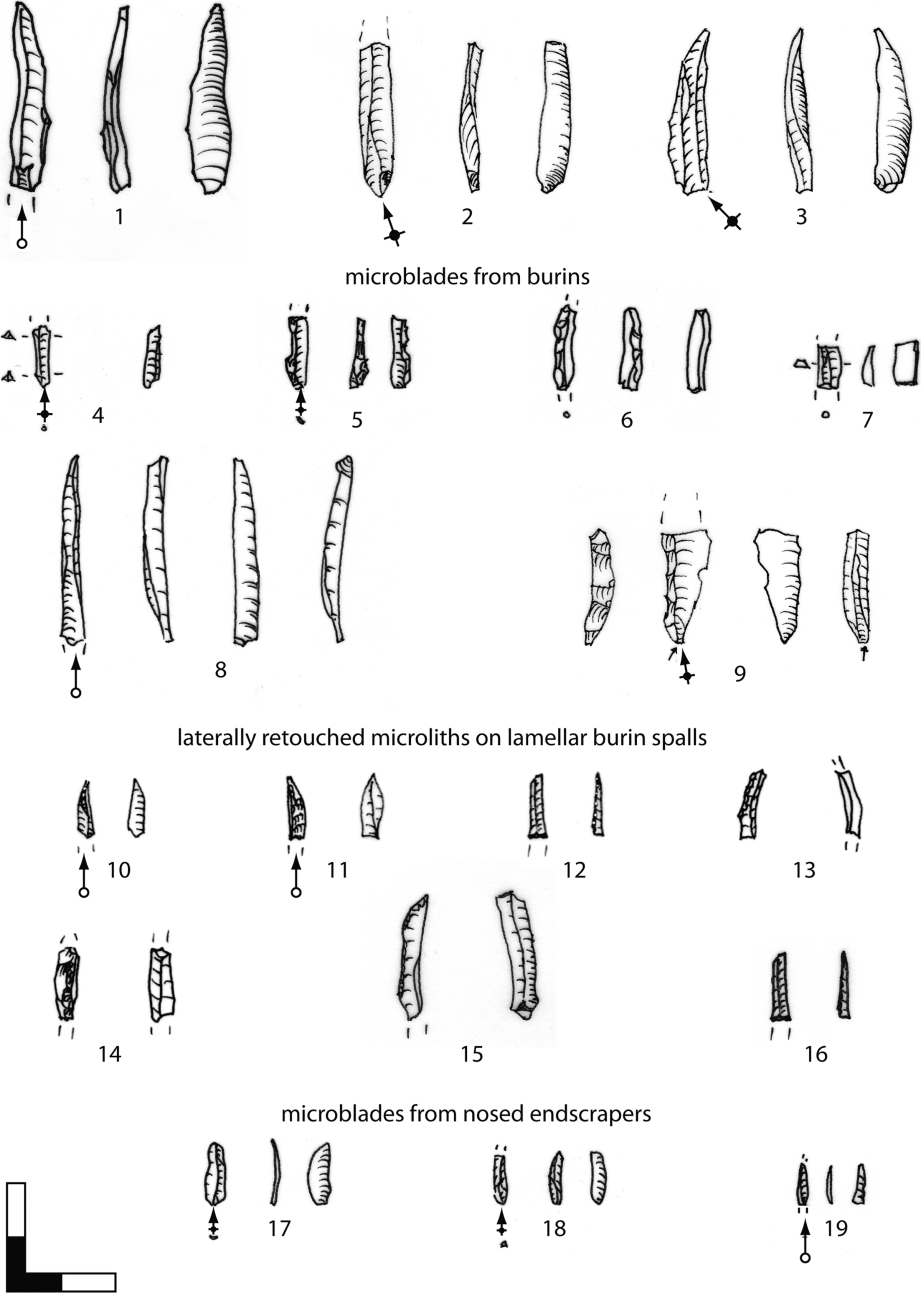
Hohle Fels, AH IV (GH 7). Target products of bladelet production: bladelets, microblades, lamellar burin spalls. 1–3: bladelets; 4–9: microblades from burins; 10: lamellar burin spall with use traces at the terminal edges from borer-like usage; 11–16: microliths on lamellar burin spalls; 17–19: microblades from carinated/nosed endscrapers; 1, 5–6, 9, 14: crested lamellar blanks. Scale 1:1. Drawings: G. Bataille.

**Fig 23:**
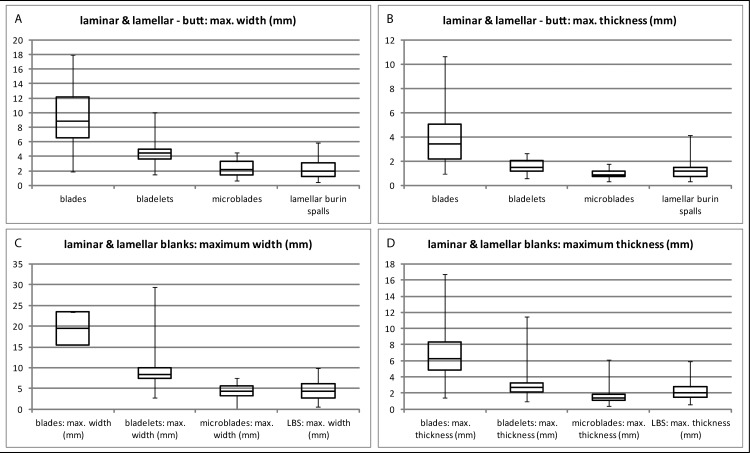
Hohle Fels, AH IV (GH 7). Laminar and lamellar blanks. (A-B) Maximum width and thickness of striking butts. (C-D) Maximum width and thickness of blanks. (A-B) Blades (n = 25), bladelets / microblades (n = 103) & lamellar burin spalls (n = 64); (C-D) Blades (n = 110), bladelets (n = 24), microblades (n = 109), lamellar burin spalls (n = 231).

Three main categories form the lamellar assemblage. These include bladelets (max. width: 7–11.99 mm), microblades (max. width: <7 mm) and lamellar burin spalls (max. width: <12 mm, usually triangular or trapezoidal cross-section, two ventral faces). Preliminary investigations of the lithic assemblage by a detailed techno-typological attribute analysis indicate that small sizes with maximum widths less than 7 mm, especially among the lamellar burin spalls, clearly dominate the assemblage (Figs 22 and 23; Table 6). Few lamellar blanks were laterally modified (Fig 22). Furthermore, knappers used other pieces in an unmodified state (Fig 22). Few pieces exhibit splintering marks at the distal tips, which probably originate from a rotating movement on hard material. In this context, the triangular cross-section of these lamellar burin spalls provides the required robustness necessary for working on hard organic objects, such as the numerous small, perforated beads.

**Table 6:** Hohle Fels, AH IV (GH 7). Laminar and lamellar blanks. Upper row: Maximum width and thickness of striking butts. Lower row: maximum width and thickness of laminar and lamellar blanks. 1: blades (bl; max. width >11.99 mm), 2: bladelets (blt; max. width 7–11.99 mm), 3: microblades (mi; max. width <7 mm), lamellar burin spalls (lbs; max. width <12 mm).

blanks: maximum values	bl: width (n = 208)	bl: thickness (n = 208)	blt: width (n = 65)	blt: thickness (n = 65)	mb: width (n = 125)	mb: thickness (n = 125)	lbs: width (n = 264)	lbs: thickness (n = 264)
**minimum (mm)**	10.87	1.44	3.63	0.94	1.2	0.41	3.82	2.06
**maximum (mm)**	42.81	16.62	11.91	11.46	9.39	6.16	11.97	5.9
**median (mm)**	19.43	7.06	8.44	2.71	4.43	1.35	3.82	2.06
**butts: maximum values**	**bl: width (n = 61)**	**bl: thickness (n = 61)**	**blt: width (n = 18)**	**blt: thickness (n = 18)**	**mb: width (n = 39)**	**mb: thickness (n = 39)**	**lbs: width (n = 77)**	**lbs: thickness (n = 77)**
**minimum (mm)**	1.88	0.94	0.47	0.035	0.65	0.35	0.47	0.34
**maximum (mm)**	17.92	10.62	14.96	7.92	4.49	1.79	5.83	4.16
**median (mm)**	8.84	3.44	4.47	1.49	2.21	0.85	1.99	1.15

## 5. Discussion

**Fig 24:**
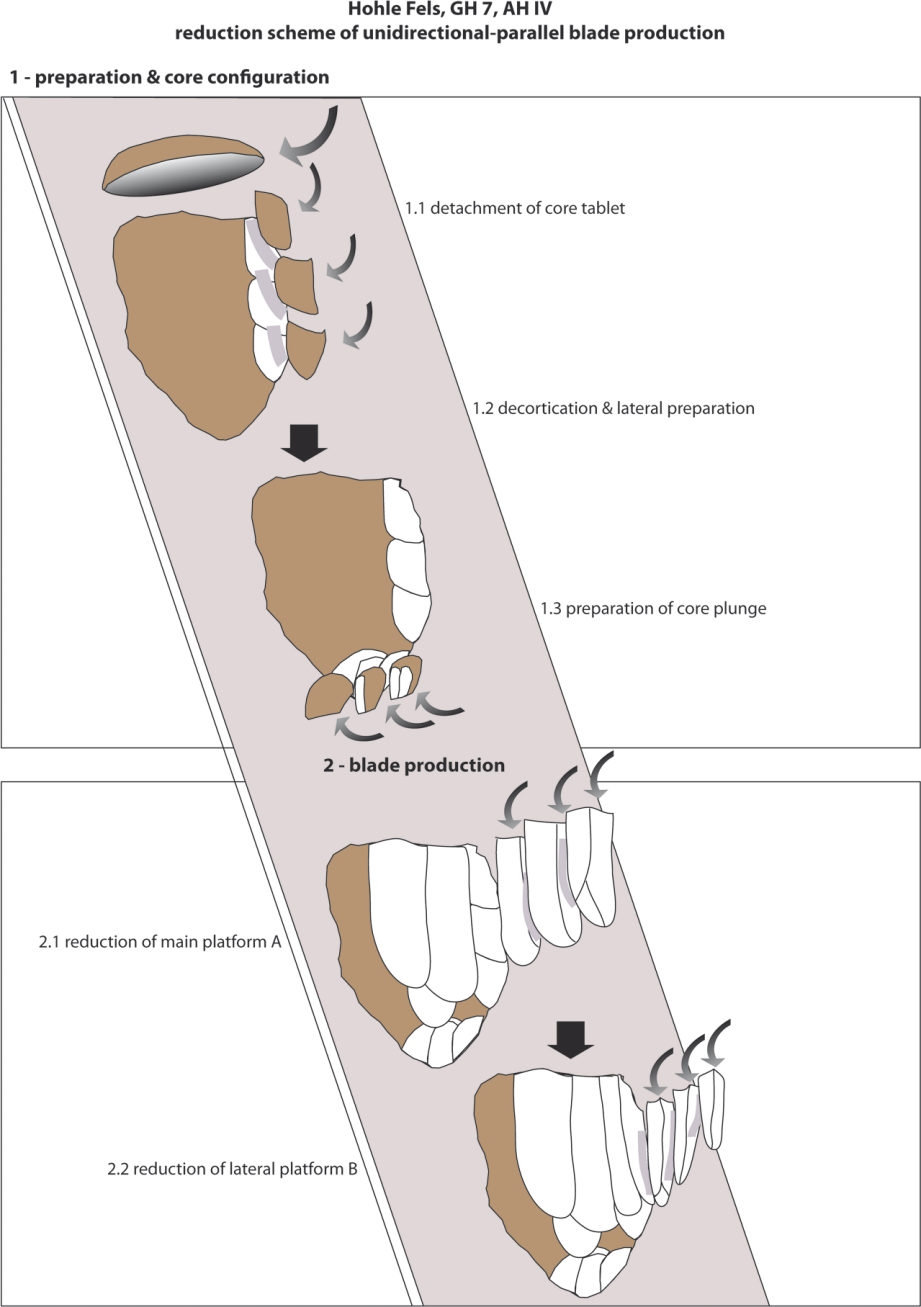
Hohle Fels, AH IV (GH 7). Synthetic reduction scheme of blade production–unidirectional-parallel method.

There are some characteristics of blade core configuration and exploitation that are diagnostic for the AH IV assemblage. One such characteristic is the preparation of flat striking surfaces by the detachment of big flakes or core tablets, thereby establishing steep reduction angles of around 60° degrees. In other cases, additional flake negatives adjust the striking angle. Another characteristic involves the intentional selection of comparatively flat round nodules deriving from the local and regional Jurassic chert („Hornstein“) sources of the Swabian Alb [33]. Usually the decortication and the formatting of the reduction face(s) is initialized from the narrower right lateral edge. According to the low quantity of cortical blanks, the major part of core preparation took place off-site. The production of target blades is conducted usually in a semi-circumferential manner. This method makes use of the broad main reduction face (A) and the narrow supplementary reduction face (B), which is commonly situated at the right core flank (Fig 24). In order to avoid overshooting, the core’s plunging termination is prepared by small blows from the lower end or the lower section of the lateral edge of the core.

Naturally, bladelet cores exhibit much smaller reduction faces than blade cores. Furthermore, in the AH IV assemblage slim reduction faces, often manufactured on blank edges, are preferred. Such reduction faces can be extremely narrow, as exemplified on the burin cores, which are a typical feature of the lithic assemblage. Reduction faces on distal or proximal ends produce short lamellar blanks and on lateral edges long lamellar blanks. Two general varieties of bladelet cores are characteristic here. The first group consists of cores with comparatively wide but short reduction surfaces. Such cores are flat, carinated and nosed endscrapers with lamellar negatives along the worked edges (Fig 17C). The second, numerically more important group is represented by different kinds of burin-cores, some of them carinated and busked. While carinated and busked cores produce mainly short products, blanks struck from regular burin-cores can be extremely narrow and thin, exhibiting high length-width ratios. Usually such burin cores show carefully prepared striking surfaces, often by truncation ("burins on truncation"), and a succession of unidirectional-parallel negatives at the lateral edge or, in some cases, at the small edges. Others are reduced as "dihedral burins." Carinated burins on blades are counted among these cores.

**Fig 25:**
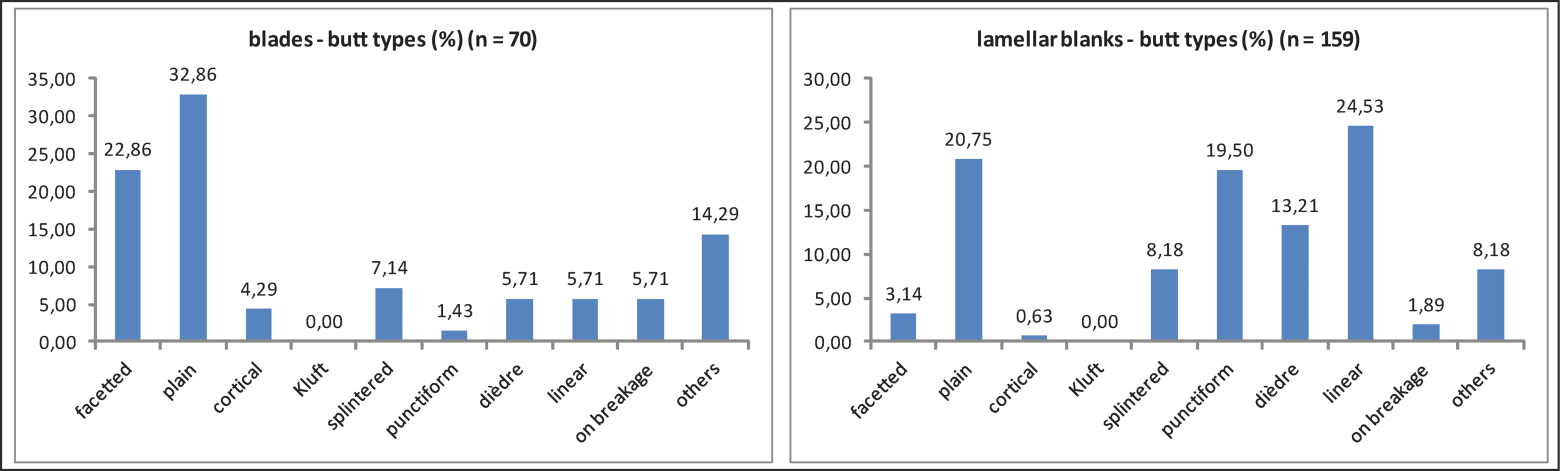
Hohle Fels, AH IV (GH 7). Laminar and lamellar blanks with preserved basal end. Butt type categories. 1. Blades (n = 70); 2. Bladelets (n = 19); 3. Microblades (N = 49); 4. Lamellar burin spalls (n = 91).

In the following, we investigate divergences and convergences between different concepts of blade and bladelet production by examining technological features such as butt type, bulbs/lips and blank profiles. Blades and bladelets are characterized by different kinds of butts that are the results of different sizes of the blanks and different knapping techniques. As indicated in the frequent plain striking platforms of cores, blades usually exhibit plain butts with median values of 8.84 mm (max. width) and 3.44 mm (max. thickness) (Fig 25; Tables 6 and 7). In contrast, lamellar blanks (bladelets, microblades and lamellar burin spalls) exhibit plain or slightly prepared butts, but are much smaller in size, with median values of 1.99–4.47 mm (width) and 0.85–1.49 mm (thickness). Accordingly, pointed and linear butt types dominate among bladelets, microblades and lamellar burin spalls. The higher share of facetted butts as well as the larger butts among the bladelets might indicate that some of these represent a continuum with the blade category. On the other hand, some microblades might come from reduced burin-cores. The differences in the sizes of butts between laminar and lamellar blanks are most likely the result of varying blank sizes but also of diverging striking techniques.

**Table 7:** Hohle Fels, AH IV (GH 7). Laminar and lamellar blanks. Butt type categories. 1: blades (bl; max. width >11.99 mm), 2: bladelets (blt; max. width 7–11.99 mm), 3: microblades (mi; max. width <7 mm), lamellar burin spalls (lbs; max. width <12 mm).

butt type	bl (N)	bl (%)	blt (N)	blt (%)	mi (N)	mi (%)	lbs (N)	lbs (%)
**without butt**	138	66.35	46	70.77	76	60.80	173	65.53
**Facetted**	16	7.69	3	4.62	0	0.00	2	0.76
**Plain**	23	11.06	8	12.31	13	10.40	12	4.55
**cortical**	3	1.44	0	0.00	1	0.80	0	0.00
**Kluft**	0	0.00	0	0.00	0	0.00	0	0.00
**splintered**	5	2.40	0	0.00	2	1.60	11	4.17
**punctiform**	1	0.48	3	4.62	11	8.80	17	6.44
**dièdre**	4	1.92	1	1.54	5	4.00	15	5.68
**linear**	4	1.92	2	3.08	16	12.80	21	7.95
**on breakage**	4	1.92	0	0.00	0	0.00	3	1.14
**others**	10	4.81	2	3.08	1	0.80	10	3.79
**Total**	208	100	65	100	125	100	264	100

**Fig 26:**
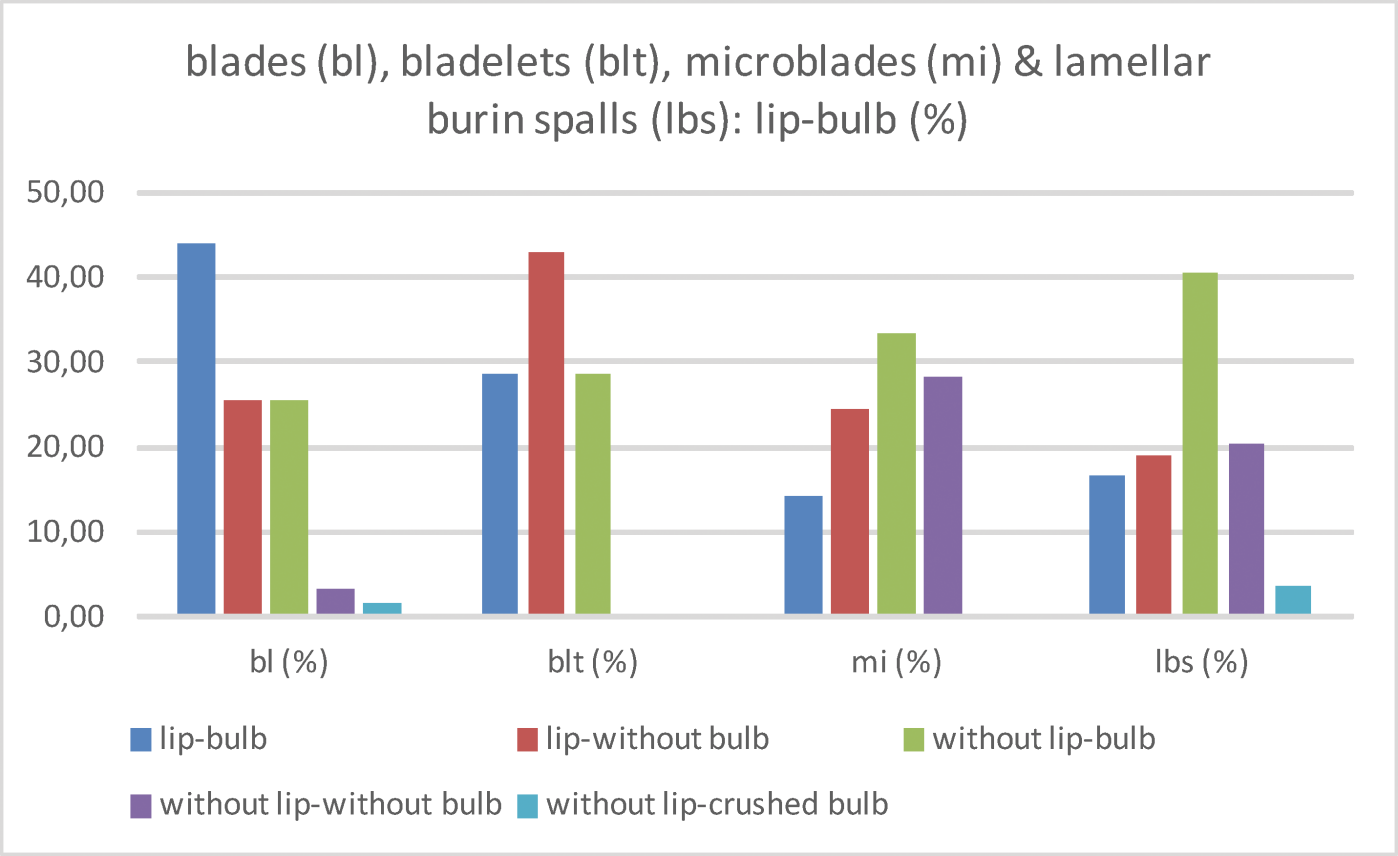
Hohle Fels, AH IV (GH 7). Laminar and lamellar blanks with preserved bulbs and lips. Bulbs and lips. Blades (n = 59); bladelets (n = 17); microblades (n = 57); lamellar burin spalls (n = 84).

This technological and morphological dichotomy is also observed if one considers combined bulbs and lips and the presence or absence of bulbar scars („Schlagnarbe“) or "esquillements du bulbe," which have been discussed as specific features in the use of soft mineral hammers [34, 35]. Moreover, this technological feature has also been connected to the punch technique [36, 37]. More recent experimental investigations have challenged the distinction between hard and soft mineral hammer as well as organic hammer [38, 39]. In the Hohle Fels assemblage, bulbar scars are rare, and weak *esquillements de bulbe* solely occur on laminar but not lamellar blanks (Figs 15 and 22). Moreover, pronounced bulbs occur only occasionally and more often on blades than on lamellar blanks. Likewise, in other Aurignacian horizons such as Siuren 1 (Crimea) or Kostenki 14, “ash layer” (Central Russian Plain) [19, 20, 40], the combined occurrence of weak lips and bulbs is a characteristic feature of the blank assemblage (Fig 26; Table 8). Though the differences in these technological features are moderate with regard to laminar and lamellar blanks, clear differences are present in contrast to Middle Palaeolithic blank assemblages, which usually feature pronounced bulbs and no lips. In AH IV, the combination of weak lips and weak bulbs is usual. Pronounced bulbs are rare and more often occur on blades and flakes than on lamellar blanks. Only lamellar blanks from burins usually lack striking lips, which might be the result of blank detachment in an orthogonal to tangential gesture. In contrast, it is likely that soft mineral retouchers were regularly used for blade/flake production while organic retouchers most likely played a role in the context of lamellar production sequences. Soft mineral striking instruments of sandstone and quarzite are present within the Aurignacian assemblages of Hohle Fels.

**Table 8:** Hohle Fels Cave, AH IV (GH 7). Laminar and lamellar blanks with preserved bulbs and lips. Bulbs and lips. 1: blades (bl; max. width >11.99 mm), 2: bladelets (blt; max. width 7–11.99 mm), 3: microblades (mi; max. width <7 mm), lamellar burin spalls (lbs; max. width <12 mm).

lip-bulb	bl (N)	bl (%)	blt (N)	blt (%)	mi (N)	mi (%)	lbs (N)	lbs (%)
**lip-bulb**	26	44.07	5	29.41	8	14.04	14	16.67
**lip-without bulb**	15	25.42	3	17.65	14	24.56	16	19.05
**without lip-bulb**	15	25.42	8	47.06	19	33.33	34	40.48
**without lip-without bulb**	2	3.39	0	0.00	16	28.07	17	20.24
**without lip-crushed bulb**	1	1.69	1	5.88	0	0.00	3	3.57
**Total**	59	100	17	100	57	100	84	100

**Fig 27:**
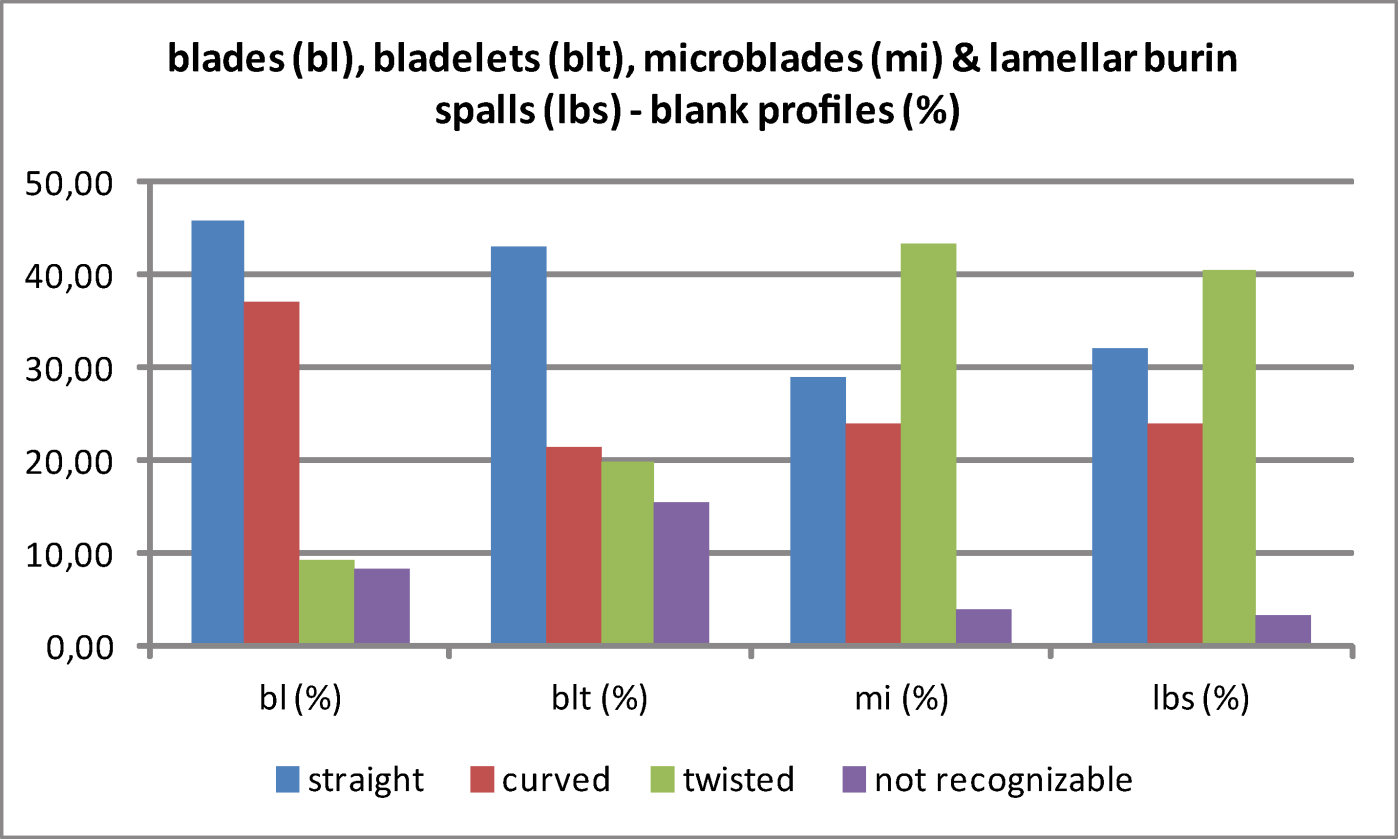
Hohle Fels, AH IV (GH 7). Laminar and lamellar blanks. Blank profiles. 1: blades (max. width >11.99 mm), 2: bladelets (max. width 7–11.99 mm), 3: microblades (max. width <7 mm), 4: lamellar burin spalls (max. width <12 mm). Blades (n = 208), bladelets (n = 65), microblades (n = 125) & lamellar burin spalls (n = 264).

Furthermore, divergent patterns in the profiles between laminar and lamellar blanks might indicate different knapping methods as well as the deviating configurations and width of the reduction surfaces (Fig 27; Table 9). Regarding blades, straight and slightly curved profiles dominate. This is due not only to the straight reduction surfaces but in all likelihood to the use of hammerstones in direct orthogonal gesture. This type of blank production results in the comparatively short but thick and wide blanks. In contrast, the bladelet assemblage is clearly dominated by twisted profiles, many of them on-axis because of the small and curved reduction faces. Moreover, bladelets were predominantly produced using tangential gesture, likely with organic retouchers. Bladelets exhibit a higher share of straight and a lower share of twisted profiles than microblades and lamellar burin spalls. Again this investigation might speak for a continuum among some of the bladelets with the blades. Especially the long and slim lamellar burin spalls exhibit on-axis twisted profiles, which are a result of the reduction along the slim, mostly lateral core edges. In contrast, a number of regular microblades show off-axis twisted profiles; this is most likely due to the fact that these pieces were struck from the edges of flat carinated and nosed endscrapers, which are part of the AH IV assemblage. Based on the blank profiles, the Hohle Fels IV assemblage differs from Roc-de-Combe assemblages, which are characterized by off-axis twisted bladelets.

**Table 9:** Hohle Fels Cave, AH IV (GH 7). Laminar and lamellar blanks. Blank profiles. 1: blades (bl; max. width >11.99 mm), 2: bladelets (blt; max. width 7–11.99 mm), 3: microblades (mi; max. width <7 mm), lamellar burin spalls (lbs; max. width <12 mm).

blank profile	bl (N)	bl (%)	blt (N)	blt (%)	mi (N)	mi (%)	lbs (N)	lbs (%)
**Straight**	95	45.67	28	43.08	36	28.80	85	32.20
**Curved**	77	37.02	14	21.54	30	24.00	63	23.86
**Twisted**	19	9.13	13	20.00	54	43.20	107	40.53
**not recognizable**	17	8.17	10	15.38	5	4.00	9	3.41
**Total**	208	100	65	100	125	100	264	100

**Fig 28:**
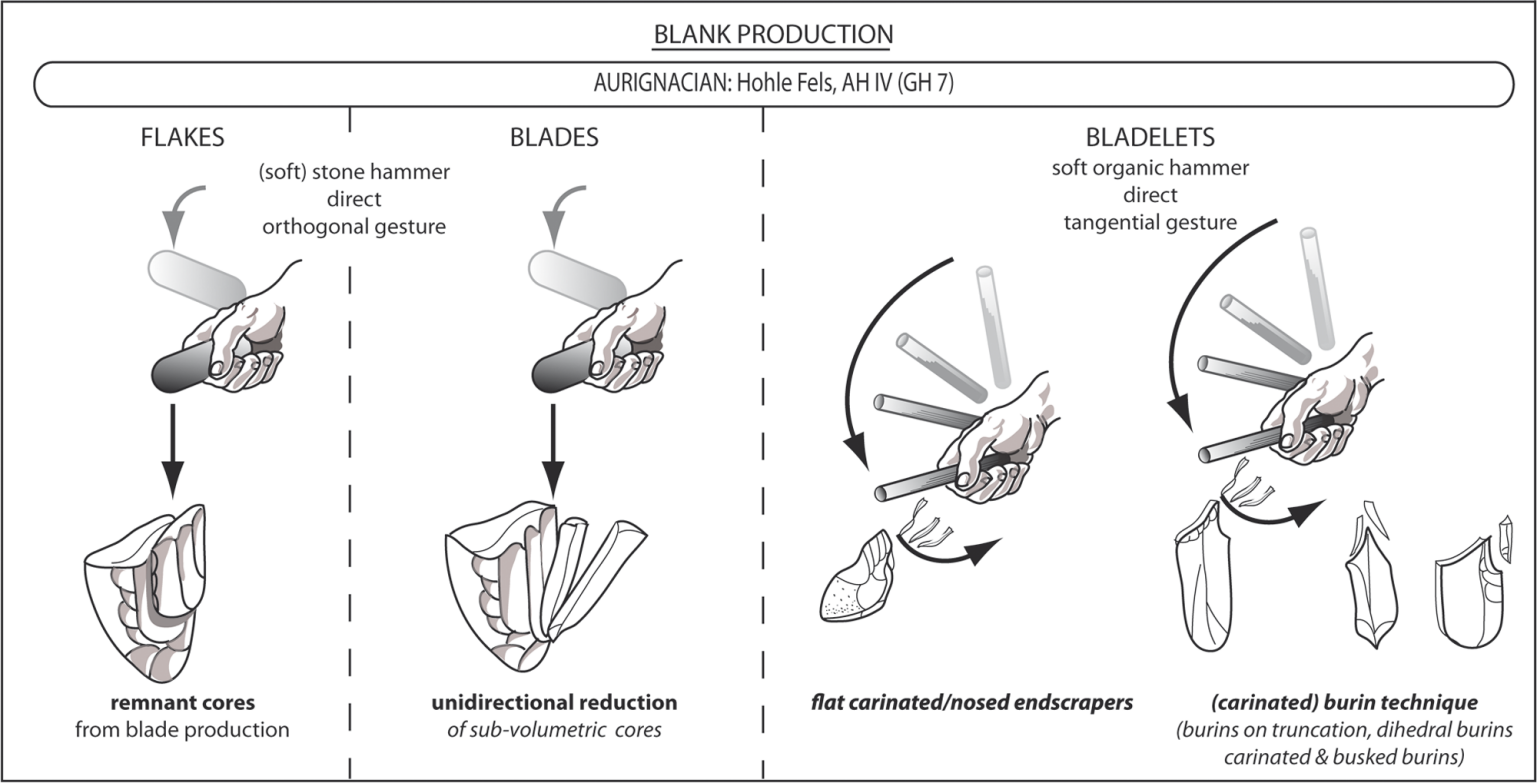
Hohle Fels, AH IV (GH 7). Technological properties of flake, blade and bladelet/microblade production.

**Fig 29:**
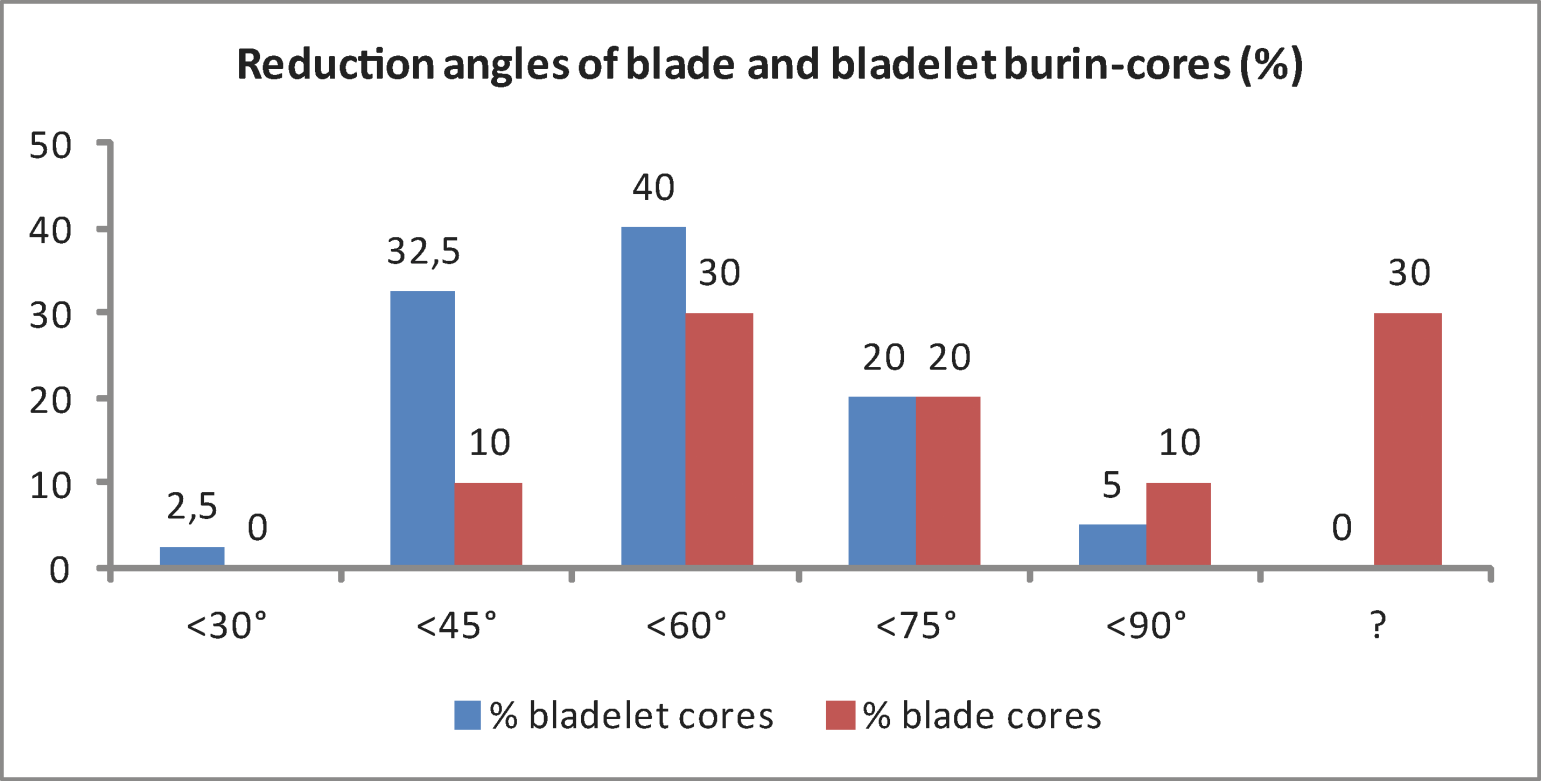
Hohle Fels, AH IV. Reduction angles of blade and burin-cores.

Based on the technological investigations of cores and blanks, the predominant striking technique and reduction strategy can be summarized as follows (Fig 28). Flake and blade cores exhibit identical technological properties. In all likelihood they were produced in an identical way: by direct percussion in more or less orthogonal gesture, predominately by the use of soft stone hammers. In contrast, bladelet cores and lamellar blanks indicate a production strategy dominated by direct percussion in a tangential gesture through the use of soft organic retouchers. Furthermore, bladelet burin-cores exhibit steeper reduction angles with a gravity around 45–60° (median: 58.88°; mean value: 60°) as compared to blade cores with a peak around 60–75° (median: 60°; mean value: 65.63°) (Table 10; Fig 29).

**Table 10:** Hohle Fels, AH IV. Reduction angles of blade and burin-cores.

reduction angle	<30°	<45°	<60°	<75°	<90°	?	total
n bladelet cores (burins)	1	13	16	8	2	0	40
% bladelet cores (burins)	2.5	32.5	40	20	5	0	100
n blade cores	0	1	3	2	1	3	10
% blade cores	0	10	30	20	10	30	100

### 5.1 Comparison of Hohle Fels AH IV with central and western European assemblages

The bladelet production from Hohle Fels AH IV features burin-cores and small carinated and nosed endscrapers. According to our investigations, carinated and busked burins are part of Hohle Fels AH IV and of the uppermost Aurignacian layer AH IIIa. Comparable bladelet core reduction methods are known from western European assemblages with carinated and busked burins, such as Abri Pataud, layers 7 and 8, Roc-de-Combe, layer 6, Trou de Renard and Maisières-Canal [28, 41, 42]. In Roc de Combe (Lot, France), the early Aurignacian (Dufour sub-type) is characterized by carinated endscraper-cores, non-twisted Dufour bladelets and thick Aurignacian blades with stepped (“Aurignacian”) retouch, while the late Aurignacian (Roc-de-Combe sub-type) is represented “by the production of twisted bladelets from carinated endscrapers and busked burins,” among them twisted Dufour bladelets [41, 43]. Unlike these assemblages, Hohle Fels IV lacks alternately and inversely retouched Dufour bladelets. Although carinated and busked burins are part of the bladelet core component, burins on (concave) truncation play an important role at Hohle Fels (Tables 2 and 3). In some cases, carinated and busked burins from AH IIIa and AH IV exhibit identical truncations, which are in fact striking platforms. Moreover, in western European Roc-de-Combe type assemblages knappers used soft hammers to produce blades [28]. Blade production at Hohle Fels IV is similar to that of Geißenklösterle [33]. Thick blades with mostly straight and slightly curved profiles were detached from unidirectional cores. The assemblages from AHs II and III of Geißenklösterle with Aurignacian retouch and carinated endscrapers have been equated with the early Aurignacian [44, 45]. Also, the tool assemblage from Hohle Fels AH IV fits well into the typological range of the Swabian Aurignacian. In Hohle Fels AH IV, burin-cores occur with two ivory Mladeč points (*Lautscher Spitzen*) and pointed blades (*Spitzklingen*). At the same time, bladelet production differs significantly from so-called early Aurignacian assemblages, with the production of narrow microblades from burin-cores characterizing Hohle Fels AH IV. A further exception from the proposed early Aurignacian of the Swabian Jura might be Bockstein-Törle AH VII, which features similar burins with core function such as carinated, busked and burins on truncation [36]. Additionally, horizons from Swabian cave-sites exhibit greater numbers of burin-cores, such as carinated and busked burins (Bocksteinhöhle, Sirgenstein AH IV and Hohlenstein-Stadel AH IV) [10, 36, 46]. Carinated and busked burins also occur in small numbers together with many carinated and nosed endscrapers in Hahn’s “*gewöhnliches Aurignacien*” (“typical Aurignacian”) [36]. On the other hand, burin-cores, which are typical for the evolved / late western European Aurignacian, occur in Sirgenstein AH IV and Bocksteinhöhle together with characteristic artifacts of the Western European early Aurignacian, such as thick carinated endscrapers (Sirgenstein IV), split-based points (Bocksteinhöhle) and Aurignacian blades (Sirgenstein IV) [46]. Furthermore, radiocarbon dates as well as the stratigraphic position of split-based points in the Swabian Jura underline the difficulty of using organic points for chronological attributions [47]. For example, split-based points, which are usually attributed to theearly Aurignacian are found in the upper Aurignacian horizon of Geißenklösterle and Vogelherd Caves, but not in the lower horizons. Instead, small and straight pencil-like points made from mammoth ivory are a typical feature of the lower Aurignacian horizons of the Swabian Jura. Hohle Fels AH IV is characterized by burin-cores that, according to the western European chronology, are associated with the evolved / late Aurignacian. Lautscher points are also found at Hohle Fels AH IV. According to L. Zotz, the Lautscher points together with split-based points are characteristic fossils of the early Aurignacian [48, 49].

Contrary to other burin types, the core function of carinated and busked pieces has often been addressed [50]. In the western European context, these burin-cores are usually associated with the evolved Aurignacian of the Roc-de-Combe sub-type [43, 51]. In Hohle Fels IV, further burin types can be regarded as bladelet cores, with dihedral burins and burins on concave truncation being among them. These pieces were regularly produced on blades and sometimes on flakes. Often the lateral edges of blanks were used as the reduction face for bladelet production. The core-function of dihedral burins and burin types with lateral reduction faces was described among others for the Middle Paleolithic of Riencourt-lès-Bapaume (France) [52], the initial Upper Paleolithic of Siberia [23], the Western European late / evolved Aurignacian of Abri Pataud, Level 8 and Caminade Est (France) as well as Maisiéres-Canals and Trou du Renard (Belgium) [26, 28, 42, 53], the Aurignacian sensu lato of Kostenki 14/IVb1-2 (Central Russia) [19] and the evolved Aurignacian of Siuren 1 (Crimea) [40, 54].

The Aurignacian of the Swabian Jura was described as early Aurignacian on the grounds of techno-typological investigations of the Geißenklösterle sequence [55]. Some researchers have pointed out a strong conformity among Aurignacian assemblages of the region without identifying a regional chronological development of the technocomplex [36, 46]. This paper demonstrates that horizon IV of Hohle Fels deviates from the so-called Early Aurignacian of Geißenklösterle. This is due to the high proportion of burin-cores in AH IV from which knappers struck narrow microblades, which they further modified by lateral dorsal retouch. Judging from published descriptions of Swabian Aurignacian sites, assemblages from Sirgenstein Cave (AH IV), Bockstein-Törle (AH VII) and Hohle Fels (AH IIIa) show the closest affinity to Hohle Fels AH IV [11, 36].

The technological deviation of the Hohle Fels AH IV lithic assemblage from the overall picture of the “Swabian Aurignacian” is primarily due to the high share of burin-cores and the high number of narrow triangular lamellar burin spalls with straight as well as on- and off-axis twisted profiles.

When comparing the AH IV assemblage with the western European reference stratigraphies, several dissimilarities emerge. The stratigraphy of Abri Pataud exhibits three Aurignacian phases (early, middle and late). Layer 8 (*Aurignacien évolué*) includes different burin types, among them carinated burins as well as carinated and thick nosed endscrapers [53]. Unlike Hohle Fels AH IV, Dufour bladelets with alternate and inverse retouch are present. Also the tool and core types in AH IV described above which can be associated with different western European chronological stages indicate that the stratigraphy of Abri Pataud is not always applicable as reference for Central Europe. For example, the upper Aurignacian complex of Geißenklösterle AH II is characterized by a mix of techno-typological index fossils correlated to different western European stages. Split-based organic points (Aurignacien I) co-occur with carinated and nosed endscrapers (Aurignacien I-II) and with busked burins (Aurignacien II-IV).

Considering the range of different types of bladelet cores, the Hohle Fels IV assemblage shows similarities with different Aurignacian stages of the Western European chronological system and with other UP technocomplexes. Burin cores represent the most abundant class of cores. This is consistent with the Western European late/ evolved Aurignacian, while the few carinated and nosed endscrapers are usually associated with stages I and II of the Western European Aurignacian. “Regular” burin types with lateral reduction edges, such as burins on truncation, on breakage and dihedral burins, play an important role in Hohle Fels. Moreover, AH IV is chronologically parallel to the early Aurignacian of Geißenklösterle, but older than most of the western European Roc-de-Combe Aurignacian sites. An exception might be the evolved Aurignacian from Maisières-Canal (Belgium) which chronologically ranges around 36–37 ka calBP [28].

The Hohle Fels example highlights technological and typological variability that deviates from the western European chronological model [56]. In Hohle Fels AH IV, knappers usually struck straight as well as on and off-axis twisted, narrow bladelets from cores with small reduction surfaces. These blanks deviate from those of the Roc-de-Combe sub-type, which are described as primarily off-axis twisted. Moreover, in Hohle Fels IV lamellar burin spalls with triangular or trapezoidal cross-section and two ventral faces dominate the bladelet sample. Lateral retouch is applied on the dorsal face. In contrast to assemblages of the Roc-de-Combe sub-type, Dufour bladelets are lacking at Hohle Fels IV. Such characteristics are most likely the result of varying demands in different environmental, socio-economic and cultural contexts. For instance, the splintering of the distal edges of lamellar burin spalls through rotating movements indicate a potential connection between the production of specific lamellar blanks from specific core types and the high number of small beads with fine perforations produced on site. Knappers from AH IV struck very narrow but stabile blanks from burin-cores. Such narrow but triangular bladelets exhibit the required robustness for the last step in drilling holes into small organic beads. Contrary to burin-cores, carinated and nosed endscrapers occur in much smaller quantities in this horizon, while other AHs from the same chronological stage exhibit more carinated endscraper-cores and fewer burin-cores (e.g., Geissenklösterle, AH II). In the Aurignacian of the Swabian Jura, artefact types associated with different Aurignacian stages co-occur in one and the same assemblage. A good example is AH II of Geißenklösterle, which exhibits split-based organic points and carinated endscraper-cores (stage I) together with nosed endscraper cores (stage II) and carinated and busked burins (*Aurignacien évolué*). From a qualitative point of view, all types of bladelet cores as well as enigmatic early Aurignacian types, such as split-based and Lautscher organic points, carinated endscrapers with straight reduction faces, and “Aurignacian” and pointed blades with and without stepped retouch, occur in all chronological stages over the complete period of the Swabian Aurignacian. One example among others for the vagueness found in chronological models based on lithic typology is the occurrence of busked burins in the lower (early Aurignacian) section of Abri Pataud [53]. Additional examples which illustrate the qualitative analogies as well as the techno-typological mixing of Aurignacian stages within single assemblages have been described from other sites of the Swabian Jura [46].

### 5.2. Techno-functional definition of the Hohle Fels IV facies

The Hohle Fels IV assemblage is a technological variant of the western Central European Aurignacian. The typological analogies and the main blade reduction strategy, which is identical to the blade production from Geißenklösterle, indicate that the Hohle Fels IV variety likely does not represent a distinct cultural phase. Moreover, the early dates of the assemblage between 39 and 36 ka calBP speak against a chronological interpretation as late Aurignacian.

The Hohle Fels IV variant is a techno-functional facies of the Swabian Aurignacian. The bladelet core spectrum deviates from descriptions of the Swabian Aurignacian. From a technological point of view, its main bladelet core component differs from Geißenklösterle AH II and III which were associated with the early Aurignacien / Aurignacien I [33, 45] with Aurignacian retouch and carinated endscrapers according to de Sonneville-Bordes and Djindjan [57, 58]. On the other hand, Hahn as well as Conard and Bolus rejected the interpretation of the Geißenklösterle assemblages within the western European chronological system [33, 37, 46]. Though Hohle Fels IV exhibits typological features of the early Aurignacian, such as pointed blades and “Aurignacian” blades with stepped retouch, carinated and nosed endscraper-cores as well as Lautscher / Mladeç points, bladelets and bladelet cores are in most cases clearly atypical of this phase. The main unidirectional-parallel blade production from sub-volumetric cores in Hohle Fels is a typical feature of the Aurignacian in Swabia and the European EUP in general [19, 33]. The presence of a few unidirectional-convergent cores indicates that this method was also used. In Hohle Fels IV, blade and bladelet production differ from each other technologically. Blades are mostly straight and slightly curved in comparison to lamellar blanks. The presence of bulbs in combination with bulbar scars indicates the direct percussion with soft hammerstones in orthogonal gesture. In contrast to Geißenklösterle AH III and II, bladelets in Hohle Fels IV were mostly struck from burin-cores. Beside the extremely narrow lamellar burin spalls, sometimes modified by marginal or more invasive lateral dorsal retouch, the dominance of different types of burin cores (including carinated, busked, dihedral and truncated pieces) characterizes this assemblage. Regarding the dominance of burin-cores, Hohle Fels IV is closer to the evolved/ late Aurignacian than to the early Aurignacian of Western Europe. Nevertheless, it differs in the lack of alternating and inversely retouched Dufour bladelets of Roc-de-Combe sub-types as well as in the secondary features of the lamellar blanks. Chronologically, AH IV is comparable to the early Aurignacian AH II from Geißenklösterle Cave.

We conclude that there is a techno-functional, regionally-based Aurignacian facies found in Hohle Fels Cave, which is present in the early (AH IV, GH 7) and late phase (AH IIIa, GH 6a) of the Aurignacian stratigraphy. It is characterized by bladelet production from burin-cores, which shows similiarities to the late Aurignacian of the Roc-de-Combe sub-type from western Europe, though at the same time lacking off-axis twisted Dufour bladelets and exhibiting features known from the western European early Aurignacian. Our conclusions must be qualified by the fact that all tools and core types which are characteristic for an evolved Aurignacian also occur in smaller quantities in older or contemporaneous assemblages of the so-called Swabian early Aurignacian (e.g., carinated and busked burins) [46]. Moreover, the typological spectrum of Hohle Fels AH IV is comparable to that of the early Aurignacian horizons from Geißenklösterle and Vogelherd.

## 6. Conclusion

**Fig 30:**
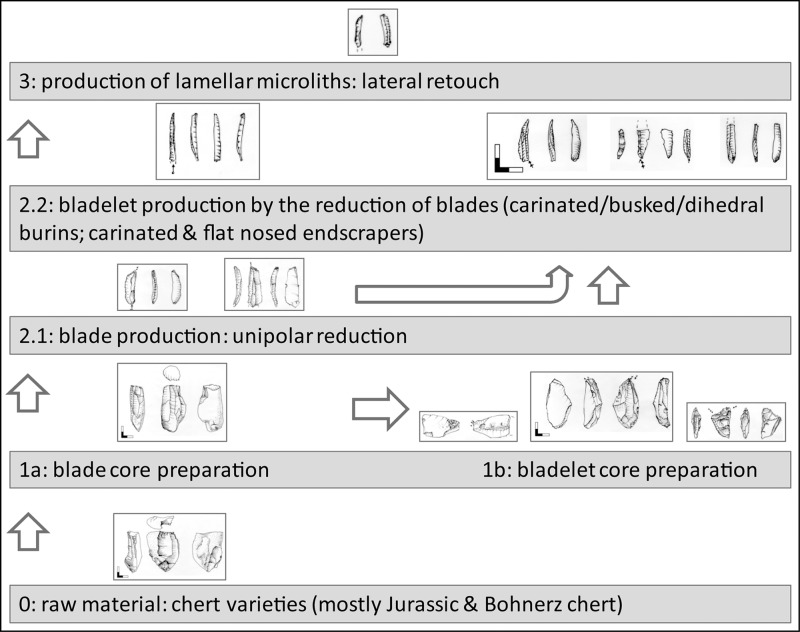
Hohle Fels, AH IV (GH 7). Operational chain of blade and bladelet production.

The technological characteristics of Hohle Fels AH IV indicate a specific technological variant for the Swabian Aurignacian described for the first time in the analysis above. With respect to the importance of bladelet production from formal burins, our assessment differs from the published descriptions of lithic artefact structure of archaeological horizons II and III of Geißenklösterle Cave in the Ach Valley and of archaeological horizons IV and V of Vogelherd Cave in the Lone Valley [45, 46]. Future research on these assemblages will need to include a comparison of laminar and lamellar production of the Swabian assemblages on a regional basis. Although blades and bladelets exhibit different technological properties, the production of lamellar burin spalls marks the last step of the operational chain, which commences with the production of parallel blades from unidirectional blade cores. In that context, unidirectional-parallel and unidirectional-convergent blade production marks the first stage and the preparation and reduction of burin-cores the second stage of the reduction sequences. In this techno-functional “Hohle Fels facies” of the Central European Aurignacian, knappers reduced unidirectional-parallel and, at times, unidirectional-convergent blade cores and regularly transformed blades into bladelet cores with steep striking platforms and narrow reduction faces (Fig 30). At the same time, the production of blades, bladelets and microblades differs technologically (Fig 23). In cases where lamellar blanks show lateral modifications, we observe that these were applied at the dorsal faces, often by marginal retouch or abrasion. Other bladelets show lateral traces of use retouch. Still others exhibit splintering at the distal tips, likely from rotating borer-like activity.

Stage 0, i.e., raw material acquisition, can be deduced from cortical remains and features of the lithic raw material brought to the site. AH IV is dominated by Jurassic chert, mostly grey in color. Bohnerz chert of reddish-gray colour is present in much smaller quantities. Knappers transformed these flat nodules into sub-prismatic blade cores. They then prepared in much smaller quantities unidirectional bladelet cores on flakes from the core preparation phase (stage 1). Next they prepared core crests as indicated by flake scars orthogonal to the main axis of blade production. The produced blades (stage 2) were further transformed (stage 3) to (a) simple tools such as endscrapers, simple or dihedral burins, borers and, above all, laterally retouched pieces. A striking characteristic of the assemblage is the transformation of blades into (b) bladelet/microblade cores (stage 2.2), as indicated by the high number of multiple burins with series of lamellar negatives. From these burin-cores the knappers struck predominantly narrow and on-axis twisted microblades. Bladelets, microblades and lamellar burin spalls with core crests suggest that striking surfaces of bladelet cores were also carefully prepared prior to blank production. A few lamellar burin spalls with intentionally retouched edges (stage 3) as well as use-wear traces attest to the intentional microblade production from burin-cores and indicate that such lamellar burin spalls were not accidental by-products of burin-tool preparation. The intentional production of lamellar blanks from burins is also supported by crested bladelets and microblades (e.g. Fig 22.9) as well as bladelet cores, among them carinated and formal burins with multiple lamellar scars (Fig 17A–17C).

The blade production found in Hohle Fels IV is comparable to the operational chain from Geißenklösterle Cave that Hahn reconstructed on the base of refitting sequences [33, 37]. Usually the testing of the raw nodule and the preparation of the striking surface commence the preparation of the core. After preparing the striking angle by detaching one big flake or a few smaller flakes, a core crest was established, or the first blade was detached along a natural ridge (“Leitgrat”). After blade production was carried out, the lateral edges and the distal part of the core was re-prepared, a sequence that is comparable to our findings from Hohle Fels AH IV. Blade core example 1 illustrates that the blade production sequences of Hohle Fels IV can be relatively short (Fig 10). In some cases knappers exploited in a similar manner the blade cores during only one reduction phase [37]. Another analogy between both operational chains is found in the reduction of flakes in the final phase of the sequences. The features of flake core example 5 (Fig 14) indicate that knappers from Hohle Fels transformed blade cores into flake cores in the final phase of reduction. Unlike what is found in the Geißenklösterle operational chain, the production in Hohle Fels of parallel blades aimed at the subsequent preparation and reduction of burin-cores. In Hohle Fels AH IV, bladelet production from burin-cores is an integrative part of the operational sequence. It is a central technological feature that bladelet production from burin-cores is intended from the beginning of the operational chain; in that context, blade production marks the initial stage of bladelet production. The Hohle Fels IV operational chain features an early reduction phase for the obtainment of thick and regular blades and a subsequent phase for the obtainment of slim bladelets and especially microblades from burin cores.

The technological repertoire of Aurignacian horizon AH IV can thus be summarized as follows:

Knappers conducted the prevailing unidirectional blade and flake production by direct impact with comparatively harder striking instruments such as soft mineral retouchers in orthogonal gesture, which is generally a typical feature for early Upper Paleolithic and Aurignacian assemblages. The use of soft stone hammers for blade and flake production was also assumed for the Eastern European Aurignacian assemblages of Siuren 1 and Kostenki 14, "layer in ash" [19, 23, 40]. Flint knappers further modified blades as tools. The recurrent use and retouch of one or both lateral edges of thick Aurignacian blades and pointed blades (Spitzklingen) could result in a stepped retouch. Knappers also chose laminar blanks to produce flat carinated and nosed bladelet cores and predominantly burin-cores with multiple laminar scars such as carinated, dihedral and burins on truncation. Moreover, they produced more massive bladelet cores on flakes from early stages of core preparation. The dominating unidirectional bladelet and microblade production was conducted by direct soft hammer percussion in tangential gesture, most likely through the use of soft organic retouchers. This striking technique was performed in combination with two diverging technological strategies:

- the production of small curved and off-axis twisted microblades from carinated/ nosed endscrapers (Fig 22.17–19)
- the production of a high share of on-axis twisted bladelets and especially microblades from burin cores (e.g. Fig 22.8).

The high number in the latter blank category, which is dominated by very tiny and slim lamellar burin spalls with straight and on-axis twisted profiles, is a specific characteristic of the Hohle Fels IV facies. Some of these microblades are laterally modified or show lateral use traces (Fig 22.11–16). Others exhibit fine splinterings on the distal tips (Fig 22.10). These traces most likely come from rotating movements on hard objects. Regarding the extraordinarily high number of small to tiny carved and pierced beads [10], we suggest that these latter microblades, which are characteristic elements of the Hohle Fels Aurignacian, were produced in order to incise such holes, thus creating fine ornaments on these objects. It is as yet unclear as to the purpose behind producing the laterally retouched microliths and unretouched microblades and if and how they were hafted. We therefore plan to conduct use-wear studies on lamellar blanks and microliths in the future in order to identify how these pieces were used. Use-wear analysis will also help to clarify whether and how sharp edges of burin-cores were additionally used as formal tools. We hope to gain a better understanding then of the functional specifics and variability of the Hohle Fels lithic assemblages.

Based on our results, we argue that the techno-functional variability of the Swabian Aurignacian is higher than what has up to now been assumed [55]. In contrast to other authors [44, 55, 59–61], we doubt the general transferability of the western European chronological system to the Central European Aurignacian record. By stressing, “*that the industrial succession “transitional”-Protoaurignacian- Aurignacian I-Aurignacian II is valid across the entire continent*, *from Romania*, *Bulgaria and Greece*, *in the East*, *to the Franco-Cantabrian region*, *in the West*” [62] the techno-typological variability resulting from varying environmental and cultural contexts is underestimated. The results presented here illustrate the contradictions that occur when one regional model is applied to another regional context. In our view, techno-typological differences resulting from varying environmental, techno-functional and cultural variables are thereby neglected. Examples for such contradictions include the qualitative co-occurrence of artefact types in Hohle Fels AH IV thought to be directory fossils of specific chronological stages, or the co-occurrence of assemblages characterized by features, which mark different chronological stages, such as the chronological contemporaneity of Geißenklösterle AH II (early Aurignacian) and Hohle Fels AH IV showing analogies with a Roc-de-Combe-like bladelet production. Conard and Bolus introduced the term *Swabian Aurignacian* to highlight the specific techno-typological characteristics of the respective assemblages [63, 64]. Moreover, by comparing techno-typological features of lithic and organic artefacts as well as personal ornaments of Aurignacian sequences at Geißenklösterle and Vogelherd, they suggested an internal regional development of the upper Aurignacian assemblages out of the lower ones [46, 63]. In the context of comparative studies and with reference to Hahn [36], Conard and Bolus state that there is little chrono-cultural variation in the Swabian Aurignacian and expressed their hesitation at “*chronostratigraphic interpretations based merely on typology*” such as in the presence or absence of tool or core types [46, 63, 64]. At the same time, we do not avoid embedding the Swabian Aurignacian in the context of a broader land use system, which encompasses further regional contextual areas. The recognition of a new techno-functional variant of the Swabian Aurignacian in AH IV should be discussed with these pre-assumptions in mind. Furthermore, a re-evaluation is necessary of the “*high degree of technological and typological homogeneity*” and variability of the Swabian Aurignacian with regard to functional, economical and chronological variables [38]. The techno-typological picture described above of the AH IV-horizon, as well as the old dates of 39–36 ka calBP, challenges the transferability of the Western European Aurignacian chronological system on Central European assemblages.

Specific artefact types within the AH IV assemblage, such as pointed blades (Spitzklingen) with at times stepped “Aurignacian” retouch, and carinated endscrapers, are seen as typical attributes of the Western European *Aurignacien ancien* [42, 46, 65]. In AH IV such artefacts co-occur with a bladelet production that presents analogies with the western European Aurignacian assemblages characterized by slim on- and off-axis twisted microblades (Roc-de-Combe sub-type) [42, 43]. In AH IV, knappers produced such lamellar blanks from core types with small reduction surfaces, especially from burin-cores. Knappers regularly prepared such cores on blades, among them carinated and busked burins. In contrast, flat carinated and nosed endscrapers prove less important. Recent investigations of pointed bladelets from Italian and French Protoaurignacian sites illustrate that the typological terms “Dufour sub-type”, “Dufour bladelets”, “Krems” and “Font-Yves points” mask a highly diverse group of retouched lamellar blanks with variable sizes, blank profiles and modes of retouch, which should be subsumed under more neutral terms, as for example alternate, direct or inverse retouched point [66]. The investigation of the co-occurrence of technological and typological attributes of different Western European Aurignacian chronological stages corroborates more recent studies from the Romanian Banat, the Crimean Peninsula, Italy and Northern Iberia [19, 20, 36, 40, 67–70]. These studies from Southwestern, Central and Eastern European settings argue for a more functional interpretation of Aurignacian techno-typological facies and varieties. Additional analyses of further Aurignacian horizons of central Europe will clarify the regional picture of the techno-typological variability. On a larger geographical scale, the potential chronological meaning of the technological variation and the impact of environmental as well as socio-cultural factors must be evaluated in future studies.
